# Diversity of *Listeria monocytogenes* Strains of Clinical and Food Chain Origins in Belgium between 1985 and 2014

**DOI:** 10.1371/journal.pone.0164283

**Published:** 2016-10-10

**Authors:** S. Bertrand, P. J. Ceyssens, M. Yde, K. Dierick, F. Boyen, J. Vanderpas, R. Vanhoof, W. Mattheus

**Affiliations:** 1 Section of Bacterial Diseases, NRC *Listeria*, Scientific Institute of Public Health, Brussels, Belgium; 2 Section of Foodborne Pathogens, NRL *Listeria monocytogenes*, Scientific Institute of Public Health, Brussels, Belgium; 3 Department of Pathology, Bacteriology and Avian Diseases, Faculty of Veterinary Medicine, Ghent University, Merelbeke, Belgium; 4 Medical Microbiology Laboratory, Scientific Institute of Public Health, Brussels, Belgium; University Medical Center Utrecht, NETHERLANDS

## Abstract

Listeriosis is a rare but severe disease, mainly caused by *Listeria monocytogenes*. This study shows the results of the laboratory-based surveillance of Listeriosis in Belgium over the period 1985–2014. Besides the incidence and some demographic data we present also more detailed microbiological and molecular characteristics of human strains isolated since 2000. The strains from the latter period were compared to food and animal strains from the same period. Our study shows that different food matrices were commonly contaminated with *L*. *monocytogenes* presenting the same PFGE profile as in patient’s isolates. Since 1985, we observed a significant decrease in incidence of the Materno-Neonatal cases (from 0.15 to 0.04 cases /100,000 inhabitants-year), which is probably to be attributed to active prevention campaigns targeting pregnant women. Despite the strengthening of different control measures by the food industry, the incidence of non-Materno-Neonatal listeriosis increased in Belgium (from 0.3 to 0.7 cases /100,000 inhabitants-year), probably due to the rise of highly susceptible patients in an aging population. This significant increase found in non-Materno-Neonatal cases (slope coefficient 7.42%/year, P<0.0001) can be attributed to significant increase in incidence of isolates belonging to serovars 1/2a (n = 393, slope coefficient 6.62%/year, P<0.0001). Although resistance to antimicrobials is rare among *L*. *monocytogenes* isolates, a trend to increasing MIC values is evident with chloramphenicol, amoxicillin, tetracycline and ciprofloxacin. We show that fluoroquinolone resistance is not linked to chromosomal mutations, but caused by a variety of efflux pumps. Our study also shows that huge majority of known underlying pathologies (426 out of 785 cases) were cancers (185/426, 43.1%) and haematological malignancies (75/185, 40.5%). Moreover the risk population is susceptible to low levels of contamination in food stressing the need of prevention campaigns specifically targeting these persons.

## Introduction

*Listeria monocytogenes* is an ubiquitous bacterium, able to survive and replicate in harsh conditions including dry environments, low temperatures and a wide pH range. This Gram-positive pathogen can infect various animals like mammals, birds, fish and crustaceans, which can either become asymptomatic carriers or develop a clinical disease. Human are most likely to be infected through consumption of contaminated foods and/or feedstuffs [1]. Once ingested, *L*. *monocytogenes* can cause severe infections such as septicaemia, meningitis, or parenchymal brain infections with a mortality reaching up to 30% [2, 3]. The majority of the listeriosis cases occur in elderly, immunosuppressed patients, pregnant women and neonates. Although the symptoms of listeriosis in pregnant women are often mild, there is a high risk of trans-placental transmission causing miscarriage, congenital defects, preterm birth and even stillbirth [4, 5, 6].

Current chemotherapy of listeriosis is based on the synergic action of ampicillin/penicillin and aminoglycosides, while trimethoprim is applied in case of intolerance to β-lactams [6, 7]. This standard treatment regime of *Listeria* has remained unchanged for decades due to the very low resistance rates against these first-line drugs (e.g., [8, 9, 10]. Indeed, recent European studies identified less than 2% of human *L*. *monocytogenes* isolates resistant for at least one antibiotic, mainly tetracycline [11, 12]. Interestingly, in contrast to β-lactam and aminoglycosides antibiotics which greatly lose their effectiveness in the intracellular environment where *L*. *monocytogenes* thrives, fluoroquinolones (FQs) remain bactericidal and can achieve a 99.9% bacterial killing within 24 hours [13]. Despite successful use of levofloxacin in acute bacterial meningitis in humans [14] and moxifloxacin in mice [15], FQs are not recommended in the treatment of listeriosis given the increased risk of side-effects in patients with impairments of the central nervous system [16]. Recently, rising non-susceptibility against fluoroquinolones has been reported in France [11] and Switzerland [12] which might have been caused by the extensive use of FQ for the treatment of multiple infections [17].

Laboratory surveillance of *L*. *monocytogenes* is based on the classical characterization of the isolate, using biochemistry testing and serotyping. Among the 13 described serotypes of *L*. *monocytogenes*, 1/2a, 1/2b and 4b are causing 95% of the human infections [3]. Serotype 4b is responsible for 33–50% of the sporadic human cases worldwide and for the majority of foodborne outbreaks reported in Europe and North America since 1980s [18]. To date, MLST and PFGE are still considered as the gold standard for the molecular subtyping of this pathogen [19, 20]. Logically, they will be replaced by whole-genome sequencing in the foreseeable future [21].

Like in the majority of European countries, Belgian surveillance of listeriosis is based on two complementary approaches: the mandatory notification of cases and the laboratory surveillance of the strains voluntary submitted to the National Reference Center (NRC) [22]. Therefore, the Belgian NRC holds a unique position to study long-term trends (1985–2015) in listeriosis, and to describe the major characteristics of the 863 cases recorded between 2000 and 2014.

## Materials and Methods

### Definitions

A listeriosis case is considered as materno-neonatal (MN) when it involves a pregnant woman, a miscarriage, a stillbirth or a new-born infant under 28 days old. When *L*. *monocytogenes* is isolated from the pregnant woman and her newborn child, it is counted as a single case. If a case fits none of these parameters, it is considered as non-materno-neonatal (n-MN).

### Bacterial isolates

In Belgium, human strains of *Listeria monocytogenes* were isolated in clinical laboratories mostly from blood (75.8%), CSF (13.4%), (in rare cases) ascites fluid (1.6%) and perinatal samples (3.6%). In some rare cases the NRC received 2 strains from one patient, one isolated from blood and the other one from CSF. The NRC performed the analysis on both isolates. These cases were then grouped as “meningitis+ sepsis”. The strains were sent on a voluntary basis to the National Reference Center for *Listeria* for confirmation of the identification and typing. The Belgian NRC exists since 1966 and the *Listeria* isolates are collected at least since 1980 in the same way.

Furthermore, the NRC receives also a wide variety of food isolates from the National Reference Laboratory for *Listeria monocytogenes* obtained from food intoxication investigations and during food monitoring programs conducted by the Federal Agency for the safety of the Food Chain. The control of *Listeria monocytogenes* in the food chain is based on self-checking systems in the food industry, an annual laboratory monitoring of specified food products by the Federal Agency for the Safety of the Food Chain and on foodborne outbreak investigations. After a non-compliant laboratory result, the food plant is inspected and the product is withdrawn from the market.

Animal isolates were obtained from clinical cases in various animal species.

In the period 1985–2014, a total of 1,436 human *Listeria monocytogenes* isolates were collected in a Belgian surveillance program. Since 2000, by using an epidemiological inquiry form, the postal code and age of the patient, as well as information on the clinical disease and the possible underlying pathology was more systematically available (In the very rare cases with multiple co-morbidities mentioned on the inquiry form, the one with the presumably most acute character was withheld).

Strain identification was carried out with API-*Listeria* biochemical microgallery and haemolysis test (bioMérieux, France). All isolates were serotyped according to a standard protocol [23] using a commercial agglutination test (Denka Seiken, Tokyo, Japan) for somatic (O) and flagellar (H) antigens.

### Antimicrobial testing and analysis of genetic determinants of FQR

Since 2000, 737 out of 863 received isolates were tested for antimicrobial susceptibility. For all isolates, the minimal inhibitory concentration (MIC) of a panel of 10 antibiotics (ampicillin, amoxicillin, gentamicin, ciprofloxacin, streptomycin, chloramphenicol, tetracycline, vancomycin, trimethoprim/sulphamethoxazole and erythromycin) was determined on Mueller-Hinton agar plates using commercial E-test strips (BioMérieux). To study the involvement of efflux pumps in fluoroquinolone resistance, the assay for ciprofloxacin, norfloxacin and moxifloxacin was repeated with either thioridazine (12.5 mg/L), chlorpromazine (25 mg/L), verapamil (200 mg/L) or reserpine (20 mg/L) added to the growth medium. If available, EUCAST clinical breakpoints were used for interpretation. For all other antibiotics, we used previously described breakpoints [12] (S1 Table).

Genomic DNA from isolates expressing reduced susceptibility to ciprofloxacin (MIC>1μg/ml) was extracted using Bacterial Genomic Miniprep Kit (Sigma), and the sequences of quinolone-resistance determining regions (QRDR) of gyrA, gyrB, parC and parE were determined as described elsewhere [24]. All sequences were screened for SNPs in comparison to corresponding regions of FQ-sensitive clinical strains using Clustal omega [25].

### Molecular typing analyses

To investigate whether trends in annual incidence were due to sporadic cases or clustered cases Pulsed-Field Gel Electrophoresis was performed on all cultivable human isolates (N = 338) and a random selection of food isolates in conjunction with the monitoring programme of the Belgian Federal Agency for the Safety of the Food Chain (N = 242) and animal isolates (N = 22) received since 2009. PFGE analysis was done following the PulseNet protocol after DNA digestion with the *Apa*I (ThermoFisher Scientific) or *Asc*I (ThermoFisher Scientific) enzymes. Banding pattern analysis was performed with BioNumerics (Applied Math). A cluster analysis on the fingerprints obtained by one enzyme was made by means of a similarity matrix calculation using the Dice coefficient followed by a dendrogram construction using the unweighted pair group method using averages (UPGMA) as algorithm with optimization and tolerance set to 1%. A concatenated analysis of both similarity matrixes of both *Apa*I and *Asc*I fingerprints can be carried out resulting in a cluster analysis characterizing all strains on the average similarity using both methods, with a UPGMA tree as result. Isolates with indistinguishable *Apa*I and *Asc*I combined macrorestriction profiles were defined as the same pulsovar. In Belgium, there is no systematic investigation of routine cases. An epidemiological investigation is only carried out in the case of outbreak. Consequently, no epidemiological field work was performed during the surveillance period presented in the manuscript to confirm observed clusters based on the detection of identical PFGE pulsovars.

Multi-locus sequence typing (MLST) was performed on all cultivable human isolates (N = 338) received since 2009 according to the method described by Ragon *et al* [26] which is based on allelic analysis of 7 housekeeping genes. Isolates were assigned to clonal complexes according to the *Listeria* MLST website (http://www.pasteur.fr/recherche/genopole/PF8/mlst/Lmono.html).

### Data analysis and statistical tests

Annual incidence rates are calculated based on population data obtained from http://statbel.fgov.be/nl/statistieken/cijfers/bevolking/structuur/leeftijdgeslacht/belgie/

The statistical variation of the number of cases (annual global incidence) was estimated by a generalized linear model according to the Poisson regression. For this purpose, the Stat10 software was used (StataCorp. 2013. Stata Statistical Software: Release 13. College Station, TX, 77845 USA). Time series analysis for evaluation of seasonal variations was performed by calculating a centred moving average (CMA) on the trimestral frequency of *Listeria monocytogenes* [27]. Furthermore, a multiple linear regression for both seasonal variation and chronological trend was calculated for this period by using Excel, Microsoft Office 2010. To evaluate the effect of age and gender on *Listeria* risk, the number of cases were grouped by age category for each gender. The association strength within each stratum of age was measured by the incidence rate ratio (IRR) with exact Fisher test for statistical significance. The difference in global risk of infection for gender was estimated by the Mantel-Haenszel Chi^2^ test.

## Results

### Epidemiological characteristics in human listeriosis

**Fig 1:**
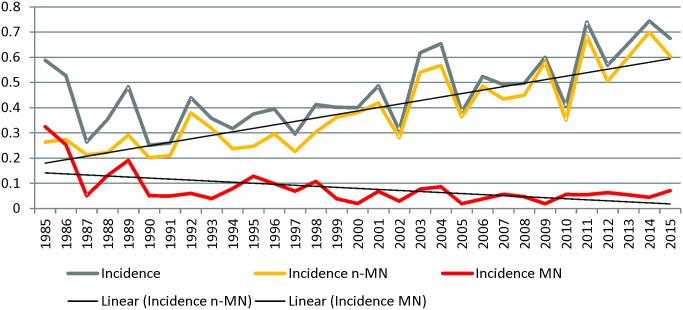
Annual incidence (/100000 inhabitants) of listeriosis, Materno-Neonatal (MN) listeriosis and non-Materno-Neonatal listeriosis in Belgium between 1985 and 2014.

**Fig 2:**
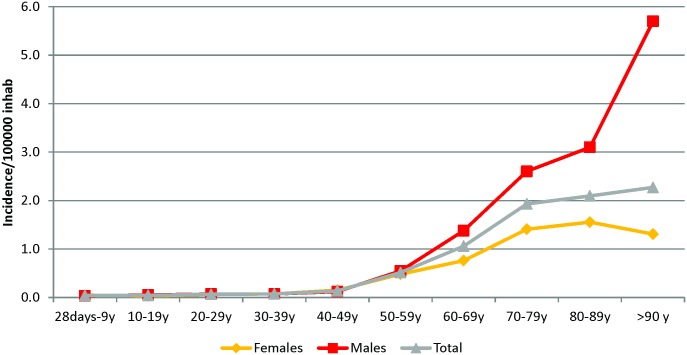
Mean incidence of non-Materno-Neonatal listeriosis per age and gender between 2000 and 2014 in Belgium.

Although it is not mandatory, the NRC *Listeria* receives almost 90% of the human *L*. *monocytogenes* isolates in Belgium. In the period 1985–2014, a total of 1,436 human *Listeria monocytogenes* isolates were collected in a Belgian surveillance program. Since 1987, the national annual incidence of listeriosis gradually increased significantly from 0.3 to 0.7 cases /100,000 inhabitants-year (slope coefficient 2.07%/year, P<0.0001; Fig 1). This increase is mainly due to the significant raise of annual incidence of n-MN listeriosis, from 0.2 to 0.7 cases/100,000 inhabitants-year (slope coefficient 3.71%/year, P<0.0001). Among the 785 n-MN forms reported since 2000, 72.2% were found in patients older than 60 years and 55.5% (436 cases on 785) were male (sex ratio of 1.3). The highest annual incidence was found in the population older than 70 years and in male older than 90 years (Fig 2). Over the same period, we noticed a significant decrease of annual incidence of the MN from 0.15 to 0.04 (slope coefficient -5.73%/year, P<0.0001). Since 2000, the annual incidence of MN form remained stable; 78 MN forms were reported comprising 16 foetal deaths or neonates deceased in the first 48 hours after birth.

Detailed analysis showed an elevated association of listeriosis with increasing age in both genders. In females, the risk almost doubles in each 10-year-age category, from less than then 8/10^7^ person-years (<39y) to greater than 80/10^7^ person-years (>80y). This effect was even more pronounced in males, with a statistically significant difference when compared to females from persons aged 60-and over (P<0.004). Moreover, when adjusted for age, the global risk of infection in males was 1.5 times greater than in females (P <0.001).

Six hundred twenty five (79.6%) patients on 785 n-MN listeriosis cases presented a septicaemia, 57 (7.3%) patients developed a meningitis and 55 (7.0%) had concomitant *L*.*monocytogenes* bloodstream infection and meningitis. Fourty five patients (5.7%) presented other symptoms like peritonitis, wound or prosthetic infections etc… Only 3 cases of the cutaneous form of listeriosis were observed, which were contracted through a professional exposure (veterinarians and farmers).

An underlying pathology was known for 426 out of 785 cases (54.3%). The huge majority of cases concerned cancers (185/426, 43.1%) and haematological malignancies (75/185, 40.5%) (Table 1).

**Table 1:** Number of n-MN listeriosis cases per associated pathologies and serotypes.

	Total	1/2a	4b	1/2b	1/2c	3a	3c	4a	4c	4d	4e	sp.
Cancer	185	92	60	19	9	2			1	1	1	0
Digestive diseases	52	23	16	13								0
Respiratory diseases	35	18	12	1	2			1			1	0
Chronic kidney diseases	33	18	11	4								0
Immunosuppression	29	17	6	3	2							1
Heart diseases	23	11	5	6								1
Iatrogenic immunosuppression	20	9	11									0
Alcoholism	20	12	5	2		1						0
Transplantation	14	6	5	2	1							0
Surgery	12	4	7	1								0
Autoimmune Diseases	2	1	1									0
infection/wound	1	1										0
No indication	359	158	146	41	6	2	1	1		1	1	2
**Total**	**785**	**370**	**285**	**92**	**20**	**5**	**1**	**2**	**1**	**2**	**3**	**4**

Concerning the seasonal trends, with the exception of the April 2011 outbreak, an increase of the sporadic cases was observed almost each year between June to November. A residual error analysis (Tarone’s test for homogeneity with Chi^2^ = 14.947 with df = 10 and P = 0.134) showed that the impact of the higher frequency in summer and autumn, in this 15-year period, is to be considered as a continuous phenomenon rather than as a coincidence.

### Strains analysis

**Fig 3:**
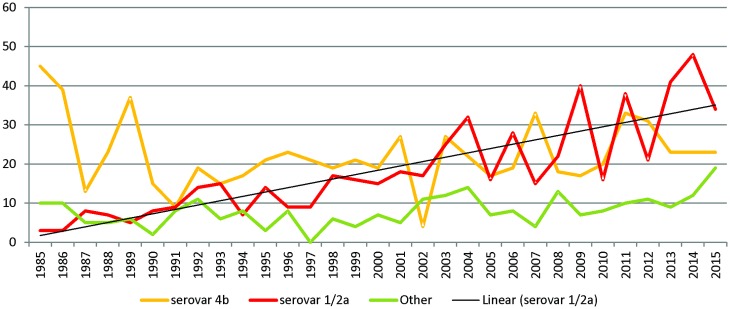
Annual incidence (/100000 inhabitants) of listeriosis caused by serovar 4b and 1/2a, and other serotypes in Belgium between 1985 and 2014.

During the period 2000–2014, 45,5% of the 863 *Listeria monocytogenes* isolated from patient were serotyped as 1/2a (n = 393), which is the dominant serotype, followed by 4b (38,3%, n = 331), 1/2b (11,5%, n = 99) and 1/2c (2,4%, n = 21). Other serotypes like 3a, 3b, 4a, 4c, 4d and 4e were rarely identified (1–5 times during this 15 years period)(Fig 3).

**Fig 4:**
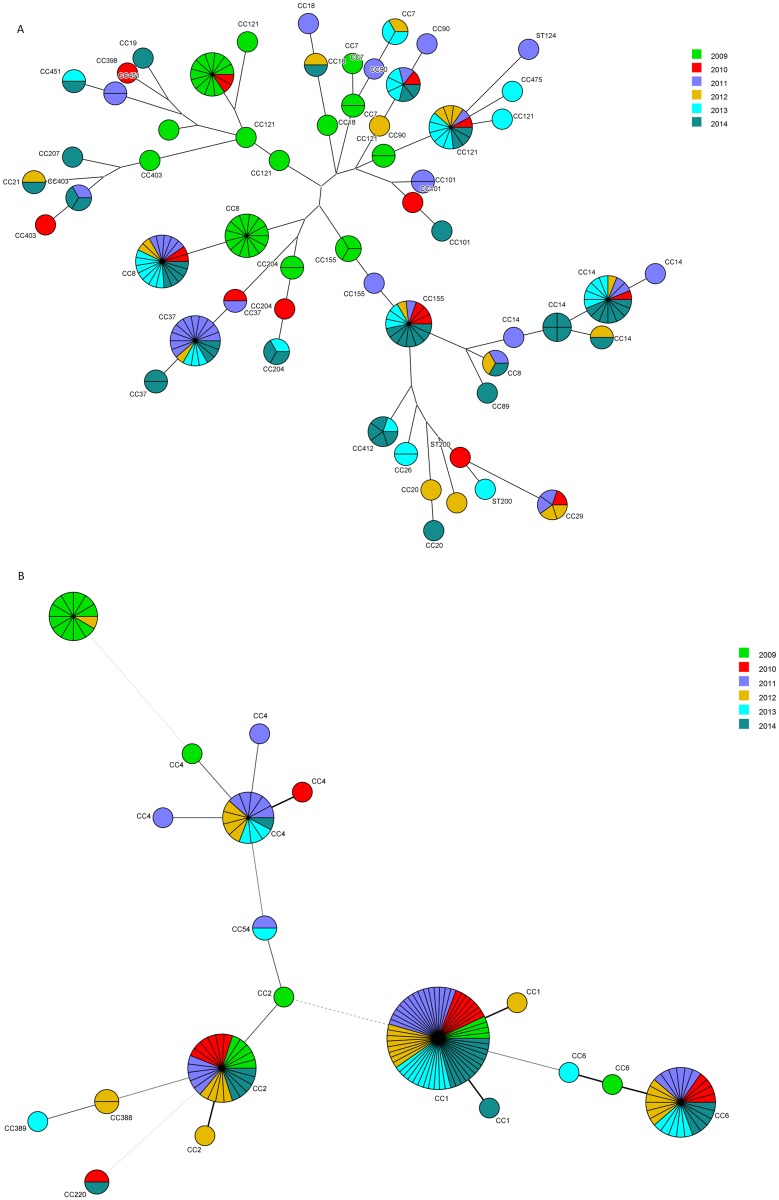
Minimal spanning tree of 7-gene MLST of *L*. *monocytogenes* isolates of serovar 1/2a (a) and 4b (b) isolated in 2009–2014. Each node represents a different 7-gene MLST profile with frequency-dependent size. Node colour represents isolation year. Branch thickness indicates number of different loci in the 7-gene MLST profile of the connected nodes.

**Fig 5:**
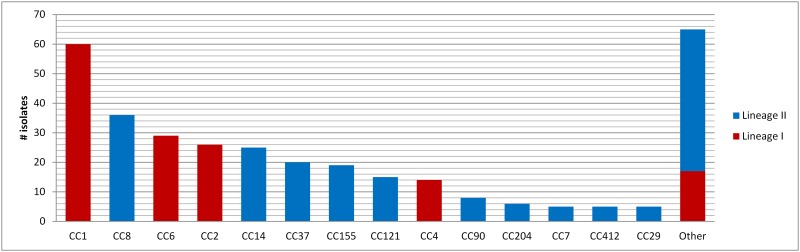
Distribution of Clonal Complexes (CC) of human *L*. *monocytogenes* isolates of 2009–2014. Rare CC observed less than 5 times are grouped (Other).

Interestingly, clear difference emerged between the two main serotypes. The significant overall increase in annual incidence of isolates belonging to serovars 1/2a (n = 393, slope coefficient 6.62%/Year, P<0.0001) can be attributed to their significant increase found in n-MN cases (slope coefficient 7.42%/Year, P<0.0001) (Fig 3). The discrete decrease in annual incidence observed in the MN cases of serovar 1/2a was not significant. We identified 77 pulsovars and 33 ST grouped in 22 clonal complexes (Figs 4a and 5). The number of sporadic 1/2a cases (unique pulsovars) remained stable, whereas the proportion of cases related to clusters corresponded with the fluctuating annual incidence during this 6-year period (Fig 3). Only one pulsovar belonging to CC8 was observed every year and often observed at high frequency (1–8 cases/year).

As shown in fig 3, the total annual incidence for serovar 4b (n = 145) isolates did not change significantly over time. However, this seems due to a significant decrease in the MN cases (slope coefficient -7.71%/year, P<0.0001), compensating the significant increase in n-MN cases (slope coefficient 1.90%/year, P<0.0001) (data not shown). We identified 58 pulsovars and 12 ST grouped in 8 clonal complexes (of which 72% (104/145) belonged to CC1, 4 or 6 defined as hypervirulent by Maury et al. (2016))[28] (Figs 4b and 5); The proportion of 4b cases related to clusters increased independent from the annual incidence (Table 2).

**Table 2:** Pulsovar diversity and cluster distribution of Listeria monocytogenes strains isolated in 2009–2014 for strains belonging to serovars 1/2a and 4b*.

	2009	2010	2011	2012	2013	2014
1/2a						
# isolates analysed	35	16	37	18	36	51
# different pulsovar	13	0	19	0	16	28
# sporadic cases	22	16	18	18	20	23
# cases in cluster	22	15	19	15	20	27
proportion in cluster	37.1	0.0	51.4	0.0	44.4	54.9
# cluster	2	0	3	0	4	7
Range of frequency each pulsovar; min-max	1–9	1–2	1–12	1–2	1–7	1–6
4b						
# isolates analysed	18	19	34	27	22	25
# different pulsovar	0	0	3	6	3	6
# sporadic cases	0.0	0.0	8.8	22.2	13.6	24.0
# cases in cluster	18	19	31	21	19	19
proportion in cluster	17	18	30	20	16	18
# cluster	0	0	1	2	1	2
Range of frequency each pulsovar; min-max	1–2	1–2	1–3	1–3	1–3	1–3

*A cluster was defined as ≥3 cases with identical pulsotypes within 14 weeks.

PFGE analysis on 175 serovar 1/2a food isolates of the same period showed 78 different pulsovars of which 22 pulsovars were identical to human pulsovars (17 out of 22 observed within a 1-year timeframe). PFGE analysis on 67 serovar 4b food isolates of the same period showed 40 different pulsovars of which 17 pulsovars were identical to human pulsovars (15 out of 17 observed within a 1-year timeframe).

Among the animal isolates, 3 (out of 6) and 12 (out of 16) pulsovars were identical to human pulsovars for serovars 1/2a and 4b, respectively.

### Antimicrobial resistance

**Fig 6:**
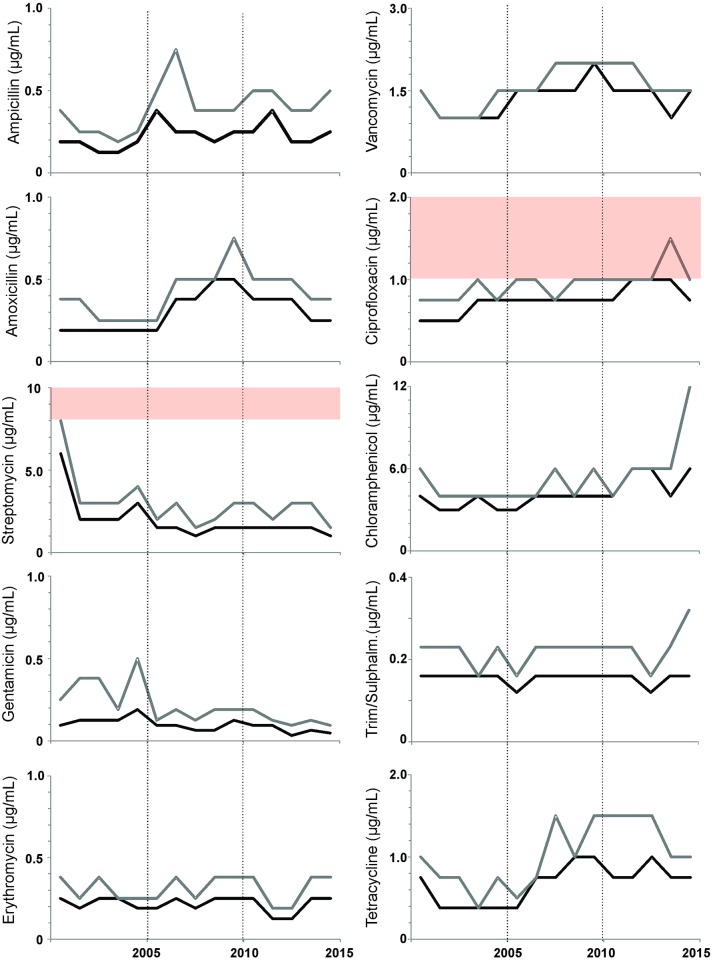
Yearly evolution of antimicrobial resistance in Belgian *L*. *monocytogenes* isolates (2000–2014). MIC_50_ and MIC_90_ values are indicated in black and grey, respectively. When in the figure range, clinical breakpoints are shown as broken lines, and zones of intermediate resistance as grey blocks.

The MIC distribution of the 10 tested antimicrobials are displayed in S1 Table, and graphically summarized in Fig 6. In 737 tested strains, phenotypic resistance to tetracycline (*n* = 2), streptomycin (*n* = 1), ampicillin (*n* = 1), chloramphenicol (*n* = 4), trimethoprim/suphamethoxazole (*n* = 1) and ciprofloxacin (*n* = 24) was detected. Only monoresistant strains were encountered, accounting for 4.5% of all isolates. Linear trend analyses (Mantel-Haenszel test) of the MIC_50_ values showed that the sensitivity to antimicrobials remained unchanged for ampicillin, vancomycin erythromycin and trimethoprim/suphamethoxazole between 2000 and 2014 or declined in case of gentamicin (P<0.0001) and streptomycin (P = 0.0005). However, a trend to increasing MIC values is evident with chloramphenicol (0.0005), amoxicillin (P<0.0001), tetracycline (P<0.0001) and ciprofloxacin (P<0.0001) (Fig 6).

Given the extraordinary potency of moxifloxacin in treatment of listeriosis, we decided to perform a molecular characterization of strains displaying fluoroquinolone resistance (S2 Table). Four main observations were made. Firstly, the four *L*. *monocytogenes* strains resistant to either ciprofloxacin and/or norfloxacin (MIC > 4μg/ml) remained susceptible to moxifloxacin (MIC < 0.5μg/ml). Secondly, this resistance was not caused by genomic target mutations, as the quinolone-resistance determining regions (QRDRs) of GyrA, GyrB, ParC and ParE in these strains were indistinguishable from corresponding regions in 13 other isolates, fully sensitive to ciprofloxacin (S2 Table). Thirdly, and in contrast to a previous study performed in France (Morgan 2010), the efflux pump inhibitor (EPI) reserpine was not able to cause a significant decrease in ciprofloxacin resistance in all cases. Indeed, while strains 2007–175 and 2011–113 displayed ≥3-fold reductions in MIC_CIP_ in the presence of reserpine, MIC_CIP_ of strain 2005–13 decreased from >32 μg/ml to only 12μg/ml, and 2006–231 was completely insensitive to reserpine (S2 Table). Fourthly, norfloxacin seems to be a less suitable substrate for the reserpine-sensitive pumps, as a maximum of 2-fold MIC_NOR_ reductions were observed.

Finally, we repeated the MIC analyses while including ½ MIC concentrations of three other EPI. We observed strong reductions in MIC_CIP/NOR_ values, but especially addition of verapamil led reversion of the resistance phenotype in all tested cases. These results strongly suggested the involvement of other, yet uncharacterized efflux pumps in fluoroquinolone resistance in isolates 2005–13 and 2006–231.

## Discussion

This study provides an overview of listeriosis trends in humans in Belgium during the last thirty years. This extensive surveillance study shows marked changes in the distribution of MN cases and n-Mn cases in this country. As reported in both other European countries and the US [4, 29, 30, 31, 32], the annual incidence of MN listeriosis cases significantly decreased from 1985 until now. This can probably be attributed to (numerous) prevention campaigns targeting pregnant women [4, 33]. Nevertheless, an active surveillance remains crucial especially in a period where immigration and /or economic migration will confront us with a vulnerable population. As shown in the study of Mook [34] an increase in the number of MN cases was noted in London affecting particularly pregnant women belonging to ethnic minorities which did not receive the food safety messages emitted by the authorities. In Belgium, the ethnic origin of the patient was not always mentioned on the inquiry form.

At the same time, our study showed that the increase of annual incidence of the n-MN listeriosis is due to the emergence of the serotype 1/2a. This increase was also recently reported in a Danish study covering the last decade [35]. These isolates remain broadly susceptible to antibiotics, although the MIC_90_ of ciprofloxacin has recently crossed the intermediate breakpoint of 1 μg/ml. Our results show that a variety of efflux pumps are responsible for this phenotype, expanding previous studies which solely linked the reserpine-dependent FepA and Lde pumps to ciprofloxacin resistance [17, 36]. The identification of underlying mechanisms will be subject on a follow-up study.

In current treatment for bacterial meningitis in adults and children, ampicillin is added to 3rd generation cephalosporin to cover *Listeria monocytogenes*. Given the low rates of resistances, there is no immediate need to change this regimen [8, 9, 10].

Within serovar 1/2a, the most frequent human pulsovar-ST8 type was the only type observed every year. This type shows relatedness with CC8 isolates responsible for outbreaks in Canada, Denmark, Switzerland and Germany, and is known to persist in food processing environments [35, 37, 38, 39]. Better adaptation to growth under stressful conditions (e.g. acidity, NaCl concentrations) and higher biofilm formation capacity may explain their persistence in food production environments [40, 41]. However, our results show that only persistence is insufficient to cause high numbers of human listeriosis cases. The frequent human pulsovar-ST8 type was observed in food during the study period but at low frequency. On the other hand, the most frequent and persistent pulsovars observed in food tended to cause few human cases. Whether this can be explained by less-than-wild-type virulence potential of these strains needs to be further investigated.

Molecular subtyping of isolates showed a higher discriminatory power of PFGE over MLST. This difference was most evident in serovar 4b where nearly four times more pulsovars than sequence types were detected (Figs 4 and 5). This can be explained by the previously reported higher recombination rate of lineage II [42], differences in gene content, mobile elements and positive selection [43]. In contrast to some reports from other European countries where the increasing annual incidence resulted from a rise in sporadic cases [44, 45], our results show that cases related to several small clusters of identical pulsovars are responsible for the observed increase in Belgium. Our study has also shown that different food products were contaminated with *L*. *monocytogenes* presenting the same pulsovar as human isolates. Even if a link can be assumed between some food products and a patient, it still remains complicated to establish a convincing circumscription of listeriosis clusters and diffuse outbreaks due to the relative long incubation period rendering it nearly impossible to establish a clear epidemiological link between the case and a common food vehicle [46]. Molecular subtyping by higher resolution techniques as Whole Genome Sequencing (WGS) might help in further investigating clusters and possible food links as has been shown in recent studies [39, 47]. However, results from Stasiewicz et al [48] showed that not necessarily all challenges associated with current subtyping techniques will be overcome by WGS and that epidemiological data will remain important.

The finding that the annual incidence of the n-MN listeriosis is higher among males over 60 years of age, was also observed in other studies [49]. A possible explanation can be found in the number of predisposing factors such as underlying diseases found to be more prevalent in males than in females [50]. Furthermore, as shown by Swaminathan [49], this increase in the annual incidence is probably largely due to the presence of highly susceptible group of patients (Cancers, immunosuppressive treatments, transplantation….) in an aging European population. These observations, combined with the success of the prevention campaigns targeting pregnant women, support the idea that similar campaigns directed to other risk groups are required to invert this trend. Another contributing factor is that the risk group (elderly, immunosuppressed patients…) seems susceptible to low levels of contamination. Indeed, during a Belgian outbreak in 2011 [46] affecting 12 persons and responsible for the death of 4 of them, the level of contamination of the hard cheese was under 100 cfu *Listeria monocytogenes*/gr. This product was thus still conform to the EC regulation. An adaptation of the EC norm (e.g. with the inclusion of food destined for patients at risk) should also be taken in consideration. In the United Kingdom, it was already recommended that food served to hospital patients should be free of *L*. *monocytogenes* [51].

In conclusion, despite the strengthening of different control measures by the food industry, the incidence of listeriosis and more specifically of n-MN listeriosis increased in Belgium as well as in other European countries during the last decade. This is probably due to the rise of highly susceptible patients (aging population developing associated diseases increasing their susceptibility to this pathogen). This issue merits a further in depth analysis. Furthermore other parameters such as the use of anti-TNF alfa, anti-acids and corticoids are known to have an impact on the increase of listeriosis [52]. Moreover, the risk population is susceptible to low levels of contamination of the food stressing the need of prevention campaign specifically targeting the persons belonging to this population.

## Supporting Information

S1 Table MIC distributions of the tested strains for the ten tested antimicrobials. Intermediate and full resistance breakpoints are indicated with broken and full lines, respectively. %NS, percentage non-susceptible isolates.
(DOCX)

S2 Table Determination of Fluoroquinolone resistance in the presence of various efflux pump inhibitors. TZ, thioridazine; CPZ, chlorpromazine; VER, verapamil, RES, reserpine.
(DOCX)

S3 Table List of human Listeria isolates used in this study with their main characteristics. (DOCX)

S4 Table List of food Listeria isolates used in this study with their main characteristics. (DOCX)

S5 Table List of veterinary Listeria isolates used in this study with their main characteristics. (DOCX)

S6 Table Seasonal repartition of Listeriosis (n-MN and MN) detected in Belgium since 2000. (N = Number of cases).
(DOCX)
