# Supplementary material for: Diversity of Listeria monocytogenes Strains of Clinical and Food Chain Origins in Belgium between 1985 and 2014
Source: PLoS One. 2016 Oct 10;11(10):e0164283. doi: 10.1371/journal.pone.0164283 (PMC5056710; doi:10.1371/journal.pone.0164283)
Supplement: S1 Table — Intermediate and full resistance breakpoints are indicated with broken and full lines, respectively. %NS, percentage non-susceptible isolates. (DOCX) [file pone.0164283.s001.docx]

|  |  |  |  |  |  |  |
| --- | --- | --- | --- | --- | --- | --- |

**Supporting Information:**

**S1 Table. MIC distributions of the tested strains for the ten tested antimicrobials.** Intermediate and full resistance breakpoints are indicated with broken and full lines, respectively. %NS, percentage non-susceptible isolates.

| **AMPICILLIN** | | **SENSTITIVE** | | | | | | | | | **RESISTANT** | | |
| --- | --- | --- | --- | --- | --- | --- | --- | --- | --- | --- | --- | --- | --- |
| **Year** | **# strains** | **0.032** | **0.064** | **0.094** | **0.125** | **0.19** | **0.25** | **0.38** | **0.5** | **0.75** | **1** | **1.5** | **%NS** |
| **2000** | 30 | 0 | 3 | 1 | 8 | 4 | 10 | 2 | 2 | 0 | 0 | 0 | 0.0 |
| **2001** | 43 | 2 | 1 | 1 | 9 | 10 | 11 | 5 | 2 | 2 | 0 | 0 | 0.0 |
| **2002** | 29 | 0 | 0 | 0 | 4 | 13 | 9 | 2 | 1 | 0 | 0 | 0 | 0.0 |
| **2003** | 26 | 0 | 1 | 4 | 12 | 5 | 3 | 1 | 0 | 0 | 0 | 0 | 0.0 |
| **2004** | 17 | 0 | 0 | 4 | 8 | 3 | 2 | 0 | 0 | 0 | 0 | 0 | 0.0 |
| **2005** | 41 | 0 | 0 | 1 | 2 | 23 | 14 | 1 | 0 | 0 | 0 | 0 | 0.0 |
| **2006** | 50 | 0 | 0 | 2 | 2 | 2 | 17 | 13 | 10 | 0 | 3 | 1 | 2.0 |
| **2007** | 46 | 0 | 0 | 2 | 2 | 14 | 7 | 12 | 2 | 5 | 2 | 0 | 0.0 |
| **2008** | 56 | 0 | 1 | 3 | 10 | 13 | 16 | 10 | 2 | 1 | 0 | 0 | 0.0 |
| **2009** | 59 | 0 | 1 | 0 | 3 | 27 | 17 | 10 | 1 | 0 | 0 | 0 | 0.0 |
| **2010** | 36 | 0 | 2 | 0 | 3 | 5 | 9 | 14 | 3 | 0 | 0 | 0 | 0.0 |
| **2011** | 75 | 1 | 2 | 1 | 4 | 17 | 23 | 17 | 10 | 0 | 0 | 0 | 0.0 |
| **2012** | 54 | 0 | 1 | 1 | 3 | 4 | 12 | 17 | 13 | 3 | 0 | 0 | 0.0 |
| **2013** | 73 | 0 | 1 | 13 | 21 | 14 | 13 | 8 | 2 | 1 | 0 | 0 | 0.0 |
| **2014** | 86 | 1 | 1 | 9 | 18 | 22 | 13 | 19 | 3 | 0 | 0 | 0 | 0.0 |

| **GENTAMICIN** | | |  | **SENSTITIVE** | | | | | | | | | | | | | | | | |  |
| --- | --- | --- | --- | --- | --- | --- | --- | --- | --- | --- | --- | --- | --- | --- | --- | --- | --- | --- | --- | --- | --- |
| **Year** |  | **0.016** | | **0.023** | **0.032** | **0.047** | **0.064** | **0.094** | **0.125** | **0.19** | **0.25** | **0.38** | **0.5** | **0.75** | **1** | **1.5** | **2** | **2.5** | **3** | **4** | **%NS** |
| **2000** | 30 | 1 | | 0 | 0 | 0 | 3 | 3 | 0 | 3 | 3 | 4 | 4 | 4 | 4 | 0 | 1 | 0 | 0 | 0 | 0.0 |
| **2001** | 43 | 0 | | 1 | 3 | 5 | 5 | 9 | 9 | 5 | 5 | 1 | 0 | 0 | 0 | 0 | 0 | 0 | 0 | 0 | 0.0 |
| **2002** | 29 | 0 | | 1 | 0 | 0 | 3 | 7 | 5 | 3 | 6 | 1 | 2 | 1 | 0 | 0 | 0 | 0 | 0 | 0 | 0.0 |
| **2003** | 26 | 0 | | 0 | 0 | 0 | 0 | 8 | 8 | 2 | 4 | 2 | 2 | 0 | 0 | 0 | 0 | 0 | 0 | 0 | 0.0 |
| **2004** | 17 | 0 | | 0 | 0 | 0 | 2 | 5 | 3 | 5 | 2 | 0 | 0 | 0 | 0 | 0 | 0 | 0 | 0 | 0 | 0.0 |
| **2005** | 41 | 0 | | 0 | 0 | 0 | 2 | 6 | 11 | 8 | 5 | 3 | 4 | 0 | 0 | 1 | 1 | 0 | 0 | 0 | 0.0 |
| **2006** | 50 | 1 | | 0 | 0 | 1 | 16 | 18 | 13 | 1 | 0 | 0 | 0 | 0 | 0 | 0 | 0 | 0 | 0 | 0 | 0.0 |
| **2007** | 46 | 1 | | 2 | 2 | 5 | 8 | 8 | 8 | 8 | 2 | 2 | 0 | 0 | 0 | 0 | 0 | 0 | 0 | 0 | 0.0 |
| **2008** | 56 | 5 | | 1 | 5 | 7 | 12 | 12 | 9 | 2 | 1 | 1 | 1 | 0 | 0 | 0 | 0 | 0 | 0 | 0 | 0.0 |
| **2009** | 59 | 4 | | 4 | 9 | 4 | 12 | 8 | 9 | 5 | 2 | 1 | 0 | 0 | 0 | 1 | 0 | 0 | 0 | 0 | 0.0 |
| **2010** | 36 | 1 | | 0 | 0 | 1 | 1 | 9 | 14 | 8 | 2 | 0 | 0 | 0 | 0 | 0 | 0 | 0 | 0 | 0 | 0.0 |
| **2011** | 75 | 1 | | 3 | 2 | 6 | 13 | 17 | 20 | 9 | 2 | 0 | 1 | 0 | 0 | 1 | 0 | 0 | 0 | 0 | 0.0 |
| **2012** | 55 | 0 | | 0 | 6 | 4 | 9 | 18 | 13 | 4 | 1 | 0 | 0 | 0 | 0 | 0 | 0 | 0 | 0 | 0 | 0.0 |
| **2013** | 73 | 12 | | 19 | 7 | 9 | 12 | 9 | 0 | 4 | 0 | 0 | 1 | 0 | 0 | 0 | 0 | 0 | 0 | 0 | 0.0 |
| **2014** | 86 | 7 | | 8 | 11 | 15 | 10 | 19 | 7 | 6 | 1 | 1 | 1 | 0 | 0 | 0 | 0 | 0 | 0 | 0 | 0.0 |

| **AMOXICILLIN** | | **SENSTITIVE** | | | | | | | | | |  |
| --- | --- | --- | --- | --- | --- | --- | --- | --- | --- | --- | --- | --- |
| **Year** | **# strains** | **0.094** | **0.125** | **0.19** | **0.25** | **0.38** | **0.5** | **0.75** | **1** | **2** | **4** | **%NS** |
| **2000** | 30 | 8 | 7 | 11 | 3 | 0 | 0 | 0 | 0 | 1 | 8 | 0.0 |
| **2001** | 43 | 3 | 3 | 18 | 10 | 8 | 0 | 0 | 1 | 0 | 3 | 0.0 |
| **2002** | 29 | 0 | 0 | 16 | 8 | 5 | 0 | 0 | 0 | 0 | 0 | 0.0 |
| **2003** | 26 | 0 | 3 | 15 | 5 | 2 | 1 | 0 | 0 | 0 | 0 | 0.0 |
| **2004** | 17 | 1 | 6 | 6 | 4 | 0 | 0 | 0 | 0 | 0 | 1 | 0.0 |
| **2005** | 41 | 2 | 9 | 23 | 7 | 0 | 0 | 0 | 0 | 0 | 2 | 0.0 |
| **2006** | 52 | 1 | 1 | 26 | 21 | 2 | 0 | 0 | 1 | 0 | 1 | 0.0 |
| **2007** | 46 | 0 | 1 | 3 | 5 | 16 | 21 | 0 | 0 | 0 | 0 | 0.0 |
| **2008** | 56 | 0 | 0 | 0 | 5 | 26 | 23 | 1 | 1 | 0 | 0 | 0.0 |
| **2009** | 62 | 0 | 0 | 0 | 2 | 27 | 28 | 5 | 0 | 0 | 0 | 0.0 |
| **2010** | 38 | 0 | 0 | 0 | 4 | 10 | 9 | 14 | 1 | 0 | 0 | 0.0 |
| **2011** | 75 | 1 | 4 | 4 | 18 | 30 | 18 | 0 | 0 | 0 | 1 | 0.0 |
| **2012** | 57 | 0 | 0 | 2 | 7 | 31 | 16 | 1 | 0 | 0 | 0 | 0.0 |
| **2013** | 73 | 0 | 0 | 7 | 27 | 23 | 16 | 0 | 0 | 0 | 0 | 0.0 |
| **2014** | 86 | 1 | 3 | 26 | 20 | 27 | 6 | 3 | 0 | 0 | 1 | 0.0 |

| **ERYTHROMYCIN** | | **SENSTITIVE** | | | | | | | |  |
| --- | --- | --- | --- | --- | --- | --- | --- | --- | --- | --- |
| **Year** | **# strains** | **0.064** | **0.094** | **0.125** | **0.19** | **0.25** | **0.38** | **0.5** | **1** | **%NS** |
| **2000** | 30 | 1 | 9 | 7 | 10 | 2 | 0 | 1 | 1 | 0.0 |
| **2001** | 43 | 0 | 0 | 3 | 11 | 18 | 9 | 2 | 0 | 0.0 |
| **2002** | 29 | 0 | 2 | 4 | 12 | 11 | 0 | 0 | 0 | 0.0 |
| **2003** | 26 | 0 | 0 | 4 | 8 | 8 | 4 | 2 | 0 | 0.0 |
| **2004** | 17 | 0 | 0 | 2 | 6 | 9 | 0 | 0 | 0 | 0.0 |
| **2005** | 41 | 0 | 2 | 9 | 21 | 7 | 2 | 0 | 0 | 0.0 |
| **2006** | 51 | 0 | 1 | 4 | 26 | 18 | 2 | 0 | 0 | 0.0 |
| **2007** | 48 | 0 | 2 | 0 | 7 | 29 | 10 | 0 | 0 | 0.0 |
| **2008** | 56 | 0 | 2 | 12 | 34 | 8 | 0 | 0 | 0 | 0.0 |
| **2009** | 61 | 0 | 0 | 2 | 26 | 26 | 7 | 0 | 0 | 0.0 |
| **2010** | 37 | 0 | 1 | 0 | 5 | 15 | 15 | 1 | 0 | 0.0 |
| **2011** | 75 | 0 | 0 | 3 | 17 | 30 | 22 | 3 | 0 | 0.0 |
| **2012** | 56 | 0 | 7 | 31 | 18 | 0 | 0 | 0 | 0 | 0.0 |
| **2013** | 73 | 1 | 9 | 34 | 22 | 7 | 0 | 0 | 1 | 0.0 |
| **2014** | 86 | 0 | 1 | 4 | 21 | 37 | 17 | 6 | 0 | 0.0 |

| **STREPTOMICIN** | | | **SENSTITIVE** | | | | | | | | | **INT. RES** | | **RES.** | |
| --- | --- | --- | --- | --- | --- | --- | --- | --- | --- | --- | --- | --- | --- | --- | --- |
| **Year** | **# strains** | **0.38** | **0.5** | **0.75** | **1** | **1.5** | **2** | **3** | **4** | **6** | **8** | **12** | **16** | **64** | **%NS** |
| **2000** | 30 | 0 | 0 | 0 | 0 | 0 | 0 | 5 | 9 | 11 | 5 | 0 | 0 | 0 | 0.0 |
| **2001** | 43 | 0 | 0 | 0 | 1 | 0 | 0 | 4 | 16 | 16 | 6 | 0 | 0 | 0 | 0.0 |
| **2002** | 29 | 0 | 0 | 0 | 0 | 3 | 18 | 7 | 1 | 0 | 0 | 0 | 0 | 0 | 0.0 |
| **2003** | 26 | 0 | 0 | 0 | 0 | 5 | 12 | 8 | 1 | 0 | 0 | 0 | 0 | 0 | 0.0 |
| **2004** | 17 | 0 | 0 | 0 | 0 | 4 | 8 | 5 | 0 | 0 | 0 | 0 | 0 | 0 | 0.0 |
| **2005** | 41 | 0 | 0 | 0 | 0 | 1 | 10 | 19 | 7 | 3 | 1 | 0 | 0 | 0 | 0.0 |
| **2006** | 50 | 0 | 1 | 4 | 19 | 16 | 6 | 2 | 1 | 0 | 1 | 0 | 0 | 0 | 0.0 |
| **2007** | 46 | 0 | 0 | 2 | 11 | 11 | 12 | 10 | 0 | 0 | 0 | 0 | 0 | 0 | 0.0 |
| **2008** | 56 | 2 | 3 | 19 | 13 | 15 | 4 | 0 | 0 | 0 | 0 | 0 | 0 | 0 | 0.0 |
| **2009** | 59 | 0 | 0 | 2 | 22 | 19 | 12 | 1 | 2 | 0 | 1 | 0 | 0 | 0 | 0.0 |
| **2010** | 36 | 0 | 1 | 2 | 3 | 12 | 13 | 4 | 1 | 0 | 0 | 0 | 0 | 0 | 0.0 |
| **2011** | 75 | 0 | 1 | 15 | 18 | 18 | 13 | 9 | 0 | 0 | 0 | 0 | 0 | 1 | 0.0 |
| **2012** | 55 | 0 | 1 | 8 | 8 | 20 | 15 | 2 | 1 | 0 | 0 | 0 | 0 | 0 | 0.0 |
| **2013** | 73 | 0 | 1 | 9 | 13 | 20 | 16 | 12 | 1 | 1 | 0 | 0 | 0 | 0 | 0.0 |
| **2014** | 85 | 2 | 4 | 7 | 17 | 21 | 17 | 10 | 4 | 2 | 1 | 0 | 0 | 0 | 0.0 |

| **TETRACYCLINE** | | | **SENSTITIVE** | | | | | | | | | **INT. RES** | | **RESISTANT** | | |
| --- | --- | --- | --- | --- | --- | --- | --- | --- | --- | --- | --- | --- | --- | --- | --- | --- |
| **Year** | **# strains** | **0.125** | **0.19** | **0.25** | **0.38** | **0.5** | **0.75** | **1** | **1.5** | **2** | **3** | **4** | **16** | **48** | **128** | **%NS** |
| **2000** | 30 | 2 | 7 | 12 | 6 | 3 | 0 | 0 | 0 | 0 | 0 | 0 | 0 | 0 | 0 | 0.0 |
| **2001** | 43 | 1 | 0 | 4 | 5 | 9 | 16 | 5 | 2 | 0 | 0 | 0 | 0 | 0 | 1 | 2.3 |
| **2002** | 29 | 1 | 1 | 6 | 9 | 6 | 5 | 1 | 0 | 0 | 0 | 0 | 0 | 0 | 0 | 0.0 |
| **2003** | 26 | 0 | 2 | 7 | 6 | 5 | 6 | 0 | 0 | 0 | 0 | 0 | 0 | 0 | 0 | 0.0 |
| **2004** | 17 | 0 | 1 | 4 | 11 | 1 | 0 | 0 | 0 | 0 | 0 | 0 | 0 | 0 | 0 | 0.0 |
| **2005** | 41 | 1 | 3 | 5 | 18 | 8 | 3 | 2 | 1 | 0 | 0 | 0 | 0 | 0 | 0 | 0.0 |
| **2006** | 58 | 4 | 4 | 7 | 19 | 20 | 3 | 1 | 0 | 0 | 0 | 0 | 0 | 0 | 0 | 0.0 |
| **2007** | 47 | 0 | 0 | 0 | 2 | 20 | 23 | 2 | 0 | 0 | 0 | 0 | 0 | 0 | 0 | 0.0 |
| **2008** | 56 | 0 | 0 | 0 | 5 | 10 | 19 | 11 | 10 | 1 | 0 | 0 | 0 | 0 | 0 | 0.0 |
| **2009** | 62 | 0 | 0 | 0 | 1 | 8 | 20 | 28 | 5 | 0 | 0 | 0 | 0 | 0 | 0 | 0.0 |
| **2010** | 38 | 0 | 0 | 0 | 0 | 1 | 12 | 16 | 8 | 1 | 0 | 0 | 0 | 0 | 0 | 0.0 |
| **2011** | 75 | 1 | 1 | 0 | 1 | 13 | 28 | 16 | 14 | 1 | 0 | 0 | 0 | 0 | 0 | 0.0 |
| **2012** | 56 | 0 | 0 | 0 | 4 | 5 | 25 | 15 | 5 | 1 | 1 | 0 | 0 | 0 | 0 | 0.0 |
| **2013** | 73 | 0 | 0 | 0 | 1 | 6 | 28 | 23 | 13 | 1 | 0 | 0 | 0 | 1 | 0 | 1.4 |
| **2014** | 86 | 0 | 3 | 1 | 0 | 13 | 39 | 22 | 8 | 0 | 0 | 0 | 0 | 0 | 0 | 0.0 |

| **VANCOMYCIN** | | **SENSTITIVE** | | | | | | |
| --- | --- | --- | --- | --- | --- | --- | --- | --- |
| **Year** | **# strains** | **0.5** | **0.75** | **1** | **1.5** | **2** | **4** | **%NS** |
| **2000** | 30 | 0 | 0 | 10 | 18 | 2 | 0 | 0.0 |
| **2001** | 43 | 0 | 1 | 9 | 32 | 1 | 0 | 0.0 |
| **2002** | 0 |  |  |  |  |  |  | 0.0 |
| **2003** | 26 | 0 | 3 | 18 | 5 | 0 | 0 | 0.0 |
| **2004** | 17 | 0 | 0 | 17 | 0 | 0 | 0 | 0.0 |
| **2005** | 41 | 0 | 0 | 21 | 20 | 0 | 0 | 0.0 |
| **2006** | 58 | 0 | 1 | 23 | 34 | 0 | 0 | 0.0 |
| **2007** | 47 | 0 | 0 | 7 | 39 | 1 | 0 | 0.0 |
| **2008** | 56 | 0 | 1 | 7 | 35 | 13 | 0 | 0.0 |
| **2009** | 61 | 0 | 0 | 1 | 49 | 11 | 0 | 0.0 |
| **2010** | 38 | 0 | 0 | 1 | 8 | 29 | 0 | 0.0 |
| **2011** | 75 | 0 | 0 | 11 | 34 | 30 | 0 | 0.0 |
| **2012** | 56 | 0 | 0 | 4 | 44 | 8 | 0 | 0.0 |
| **2013** | 73 | 0 | 0 | 15 | 58 | 0 | 0 | 0.0 |
| **2014** | 86 | 1 | 6 | 61 | 18 | 1 | 1 | 0.0 |

| **CIPROFLOXACIN** | | | **SENSTITIVE** | | | | | **INTERMEDIATE RESISTANT** | | | | **RESISTANT** | | | |  |
| --- | --- | --- | --- | --- | --- | --- | --- | --- | --- | --- | --- | --- | --- | --- | --- | --- |
| **Year** | **# strains** | **0.25** | | **0.38** | **0.5** | **0.75** | **1** | **1.5** | **2** | **3** | **4** | **6** | **8** | **16** | **32** | **%NS** |
| **2000** | 30 | 0 | | 4 | 18 | 6 | 2 | 0 | 0 | 0 | 0 | 0 | 0 | 0 | 0 | 0.0 |
| **2001** | 43 | 4 | | 9 | 22 | 8 | 0 | 0 | 0 | 0 | 0 | 0 | 0 | 0 | 0 | 0.0 |
| **2002** | 29 | 8 | | 4 | 8 | 9 | 0 | 0 | 0 | 0 | 0 | 0 | 0 | 0 | 0 | 0.0 |
| **2003** | 26 | 0 | | 4 | 10 | 9 | 3 | 0 | 0 | 0 | 0 | 0 | 0 | 0 | 0 | 0.0 |
| **2004** | 17 | 0 | | 0 | 3 | 7 | 7 | 0 | 0 | 0 | 0 | 0 | 0 | 0 | 0 | 0.0 |
| **2005** | 41 | 1 | | 6 | 11 | 19 | 3 | 0 | 0 | 0 | 0 | 0 | 0 | 0 | 1 | 2.4 |
| **2006** | 50 | 0 | | 2 | 7 | 22 | 15 | 2 | 1 | 0 | 0 | 0 | 0 | 1 | 0 | 8.0 |
| **2007** | 46 | 0 | | 2 | 6 | 26 | 11 | 1 | 0 | 0 | 0 | 0 | 0 | 0 | 0 | 2.1 |
| **2008** | 55 | 0 | | 1 | 15 | 35 | 4 | 0 | 0 | 0 | 0 | 0 | 0 | 0 | 0 | 0.0 |
| **2009** | 59 | 0 | | 3 | 23 | 25 | 8 | 0 | 0 | 0 | 0 | 0 | 0 | 0 | 0 | 0.0 |
| **2010** | 36 | 0 | | 0 | 7 | 21 | 7 | 1 | 0 | 0 | 0 | 0 | 0 | 0 | 0 | 2.7 |
| **2011** | 75 | 0 | | 0 | 12 | 41 | 20 | 1 | 0 | 0 | 0 | 1 | 0 | 0 | 0 | 4.0 |
| **2012** | 54 | 0 | | 0 | 2 | 23 | 27 | 2 | 0 | 0 | 0 | 0 | 0 | 0 | 0 | 3.7 |
| **2013** | 73 | 0 | | 0 | 0 | 20 | 50 | 3 | 0 | 0 | 0 | 0 | 0 | 0 | 0 | 4.1 |
| **2014** | 86 | 0 | | 2 | 2 | 29 | 43 | 10 | 0 | 0 | 0 | 0 | 0 | 0 | 0 | 11.6 |

| **CHLORAMPHENICOL** | | **SENSTITIVE** | | | | | | | | **INT. RES** | | **RESISTANT** | |
| --- | --- | --- | --- | --- | --- | --- | --- | --- | --- | --- | --- | --- | --- |
| **Year** | **# strains** | **0.75** | **1** | **1.5** | **2** | **3** | **4** | **6** | **8** | **12** | **16** | **32** | **%NS** |
| **2000** | 30 | 1 | 1 | 2 | 10 | 12 | 4 | 0 | 0 | 0 | 0 | 0 | 0.0 |
| **2001** | 43 | 0 | 0 | 0 | 0 | 11 | 18 | 12 | 1 | 0 | 1 | 0 | 2.3 |
| **2002** | 29 | 0 | 0 | 0 | 2 | 16 | 9 | 2 | 0 | 0 | 0 | 0 | 0.0 |
| **2003** | 26 | 0 | 0 | 1 | 4 | 9 | 11 | 1 | 0 | 0 | 0 | 0 | 0.0 |
| **2004** | 17 | 0 | 0 | 0 | 1 | 5 | 11 | 0 | 0 | 0 | 0 | 0 | 0.0 |
| **2005** | 41 | 0 | 0 | 0 | 3 | 19 | 17 | 2 | 0 | 0 | 0 | 0 | 0.0 |
| **2006** | 58 | 0 | 0 | 0 | 5 | 35 | 18 | 0 | 0 | 0 | 0 | 0 | 0.0 |
| **2007** | 48 | 0 | 0 | 0 | 0 | 7 | 38 | 3 | 0 | 0 | 0 | 0 | 0.0 |
| **2008** | 56 | 0 | 0 | 0 | 1 | 7 | 29 | 19 | 0 | 0 | 0 | 0 | 0.0 |
| **2009** | 61 | 0 | 0 | 0 | 1 | 14 | 43 | 3 | 0 | 0 | 0 | 0 | 0.0 |
| **2010** | 38 | 0 | 0 | 0 | 0 | 3 | 27 | 8 | 0 | 0 | 0 | 0 | 0.0 |
| **2011** | 75 | 0 | 0 | 0 | 1 | 36 | 38 | 0 | 0 | 0 | 0 | 0 | 0.0 |
| **2012** | 56 | 0 | 0 | 0 | 0 | 1 | 10 | 42 | 3 | 0 | 0 | 0 | 0.0 |
| **2013** | 73 | 0 | 0 | 0 | 0 | 0 | 33 | 39 | 1 | 0 | 0 | 0 | 0.0 |
| **2014** | 86 | 0 | 0 | 1 | 1 | 8 | 52 | 20 | 0 | 2 | 0 | 0 | 2.3 |

| **TRIMETHOPRIM/SULPHAMETHOXAZOLE** | | | | | **SENSTITIVE** | | | | | | **RESISTANT** | | |
| --- | --- | --- | --- | --- | --- | --- | --- | --- | --- | --- | --- | --- | --- |
| **Year** | **# strains** | **0.002** | **0.006** | **0.008** | | **0.012** | **0.016** | **0.023** | **0.032** | **0.047** | **0.06** | **0.12** | **%NS** |
| **2000** | 30 | 0 | 1 | 0 | | 4 | 19 | 5 | 0 | 1 | 0 | 0 | 0.0 |
| **2001** | 43 | 0 | 0 | 0 | | 5 | 20 | 16 | 2 | 0 | 0 | 0 | 0.0 |
| **2002** | 29 | 0 | 0 | 3 | | 4 | 13 | 9 | 0 | 0 | 0 | 0 | 0.0 |
| **2003** | 26 | 0 | 0 | 0 | | 4 | 10 | 12 | 0 | 0 | 0 | 0 | 0.0 |
| **2004** | 17 | 0 | 0 | 1 | | 6 | 10 | 0 | 0 | 0 | 0 | 0 | 0.0 |
| **2005** | 41 | 0 | 0 | 0 | | 0 | 31 | 9 | 0 | 1 | 0 | 0 | 0.0 |
| **2006** | 58 | 0 | 0 | 2 | | 31 | 23 | 2 | 0 | 0 | 0 | 0 | 0.0 |
| **2007** | 48 | 0 | 0 | 1 | | 10 | 31 | 6 | 0 | 0 | 0 | 0 | 0.0 |
| **2008** | 56 | 0 | 0 | 1 | | 9 | 23 | 22 | 0 | 0 | 0 | 1 | 1.7 |
| **2009** | 61 | 1 | 2 | 0 | | 16 | 29 | 11 | 2 | 0 | 0 | 0 | 0.0 |
| **2010** | 38 | 1 | 0 | 1 | | 9 | 20 | 7 | 0 | 0 | 0 | 0 | 0.0 |
| **2011** | 75 | 0 | 0 | 0 | | 8 | 44 | 23 | 0 | 0 | 0 | 0 | 0.0 |
| **2012** | 56 | 0 | 0 | 0 | | 2 | 25 | 28 | 1 | 0 | 0 | 0 | 0.0 |
| **2013** | 73 | 1 | 0 | 13 | | 40 | 17 | 2 | 0 | 0 | 0 | 0 | 0.0 |
| **2014** | 86 | 1 | 1 | 11 | | 23 | 30 | 14 | 4 | 2 | 0 | 0 | 0.0 |
