# Supplementary material for: Diversity of Listeria monocytogenes Strains of Clinical and Food Chain Origins in Belgium between 1985 and 2014
Source: PLoS One. 2016 Oct 10;11(10):e0164283. doi: 10.1371/journal.pone.0164283 (PMC5056710; doi:10.1371/journal.pone.0164283)
Supplement: S2 Table — TZ, thioridazine; CPZ, chlorpromazine; VER, verapamil, RES, reserpine. (DOCX) [file pone.0164283.s002.docx]

| **S2 Table. Determination of Fluoroquinolone resistance in the presence of various efflux pump inhibitors.** TZ, thioridazine; CPZ, chlorpromazine; VER, verapamil , RES, reserpine. | | | | | | | | | | | | | | | | | | | | |
| --- | --- | --- | --- | --- | --- | --- | --- | --- | --- | --- | --- | --- | --- | --- | --- | --- | --- | --- | --- | --- |
| **No. NRC** | | **Ciprofloxacin (MIC)** | | | | | **Norfloxacin (MIC)** | | | | | **Moxifloxacin (MIC)** | | | | | **QRDR sequencing** | | | |
|  |  | **Control** | **TZ** | **CPZ** | **VER** | **RES** | **Control** | **TZ** | **CPZ** | **VER** | **RES** | **Control** | **TZ** | **CPZ** | **VER** | **RES** | **gyrA** | **gyrB** | **parC** | **parE** |
| **Resistant isolates:** | | |  |  |  |  |  |  |  |  |  |  |  |  |  |  |  |  |  |  |
|  | 2005-13 | **>32** | 3 | 2 | 1 | 12 | **64** | 6 | 6 | 2 | 48 | 0.5 | 0.25 | 0.25 | 0.25 | 0.38 | wt | wt | wt | wt |
|  | 2006-231 | **24** | 2 | 2 | 1 | 24 | **64** | 6 | 6 | 1.5 | 32 | 0.38 | 0.25 | 0.25 | 0.25 | 0.25 | wt | wt | wt | wt |
|  | 2007-175 | **16** | 1 | 1 | 0.5 | 2 | 4 | 2 | 3 | 2 | 3 | 0.19 | 0.19 | 0.19 | 0.19 | 0.19 | wt | wt | wt | wt |
|  | 2011-113 | **6** | 0.75 | 0.5 | 0.5 | 2 | **16** | 4 | 2 | 2 | 8 | 0.25 | 0.19 | 0.125 | 0.25 | 0.25 | wt | wt | wt | wt |
| **Control strains:** | | |  |  |  |  |  |  |  |  |  |  |  |  |  |  |  |  |  |  |
|  | S13BD04073 | 0.75 | 0.5 | 0.5 | 0.38 | 0.5 | 4 | 2 | 2 | 2 | 2 | 0.38 | 0.25 | 0.38 | 0.38 | 0.25 | wt | wt | wt | wt |
|  | 2007-99 | 0.75 | 0.38 | 0.5 | 0.5 | 0.38 | 4 | 1.5 | 1.5 | 1.5 | 2 | 0.19 | 0.125 | 0.19 | 0.19 | 0.19 | wt | wt | wt | wt |
|  | 2009-133 | 0.5 | 0.25 | 0.38 | 0.5 | 0.38 | 3 | 1.5 | 1.5 | 2 | 2 | 0.25 | 0.25 | 0.19 | 0.25 | 0.19 | wt | wt | wt | wt |
|  | 2009-175 | 0.75 | 0.5 | 0.75 | 0.75 | 0.75 | 4 | 2 | 2 | 3 | 3 | 0.25 | 0.25 | 0.25 | 0.25 | 0.25 | wt | wt | wt | wt |
|  | 2009-238 | 0.5 | 0.38 | 0.38 | 0.38 | 0.5 | 2 | 1.5 | 1.5 | 1.5 | 1.5 | 0.25 | 0.19 | 0.25 | 0.25 | 0.19 | wt | wt | wt | wt |
|  | 2010-054 | 1 | 0.38 | 1 | 1 | 0.75 | 6 | 2 | 3 | 3 | 4 | 0.25 | 0.25 | 0.25 | 0.25 | 0.19 | wt | wt | wt | wt |
|  | 2008-68 | 0.75 | 0.5 | 0.75 | 0.75 | 0.5 | 2 | 1.5 | 1.5 | 2 | 2 | 0.25 | 0.25 | 0.25 | 0.25 | 0.25 | wt | wt | wt | wt |
|  | 2008-114 | 1 | 0.5 | 0.5 | 0.75 | 0.75 | 1.5 | 1.5 | 1.5 | 1.5 | 1.5 | 0.25 | 0.25 | 0.25 | 0.25 | 0.19 | wt | wt | wt | wt |
|  | 2008-158 | 0.75 | 0.5 | 0.5 | 0.75 | 0.75 | 2 | 1.5 | 1.5 | 1.5 | 2 | 0.25 | 0.19 | 0.25 | 0.25 | 0.19 | wt | wt | wt | wt |
|  | 2008-209 | 0.5 | 0.5 | 0.75 | 0.75 | 0.5 | 2 | 2 | 2 | 2 | 2 | 0.19 | 0.19 | 0.19 | 0.19 | 0.125 | wt | wt | wt | wt |
|  | 2007-174 | 0.5 | 0.38 | 0.5 | 0.5 | 0.5 | 1.5 | 1 | 1 | 1.5 | 1.5 | 0.19 | 0.125 | 0.19 | 0.19 | 0.19 | wt | wt | wt | wt |
|  | S14BD05525 | 0.5 | 0.25 | 0.5 | 0.38 | 0.38 | 1.5 | 1 | 1 | 1 | 1.5 | 0.125 | 0.125 | 0.125 | 0.125 | 0.125 | wt | wt | wt | wt |
|  | S14BD01292 | 0.5 | 0.125 | 0.19 | 0.25 | 0.25 | 1.5 | 0.5 | 0.75 | 1 | 1 | 0.125 | 0.064 | 0.094 | 0.094 | 0.094 | wt | wt | wt | wt |
