# Supplementary material for: Diversity of Listeria monocytogenes Strains of Clinical and Food Chain Origins in Belgium between 1985 and 2014
Source: PLoS One. 2016 Oct 10;11(10):e0164283. doi: 10.1371/journal.pone.0164283 (PMC5056710; doi:10.1371/journal.pone.0164283)
Supplement: S3 Table — (DOCX) [file pone.0164283.s003.docx]

**S3 Table. List of human Listeria isolates used in this study with their main characteristics**

| **ID N°** | **serotype** | **Year** | **Form** | **Symptoms** | **Associated pathologies** |
| --- | --- | --- | --- | --- | --- |
| 5 | 1/2a | 2000 | n-MN | Meningitis | Chronic kidney diseases |
| 8 | 4b | 2000 | n-MN | Sepsis | No indication |
| 12 | 4b | 2000 | n-MN | Sepsis | Cancer |
| 13 | 4b | 2000 | n-MN | Meningitis | No indication |
| 14 | 1/2a | 2000 | n-MN | Meningitis | No indication |
| 16 | 1/2c | 2000 | MN | Meningitis + sepsis | No indication |
| 17 | 1/2b | 2000 | n-MN | Meningitis | No indication |
| 18 | 4b | 2000 | n-MN | Sepsis | Cancer |
| 26 | 1/2b | 2000 | n-MN | Unknown | No indication |
| 30 | 4b | 2000 | n-MN | Meningitis | No indication |
| 42 | 1/2b | 2000 | n-MN | Sepsis | No indication |
| 43 | 1/2b | 2000 | n-MN | Sepsis | Alcoholism |
| 48 | 1/2a | 2000 | n-MN | Sepsis | Cancer |
| 55 | 4b | 2000 | n-MN | Sepsis | Cancer |
| 59 | 1/2b | 2000 | MN | Unknown | No indication |
| 74 | 4b | 2000 | n-MN | Sepsis | No indication |
| 75 | 1/2a | 2000 | n-MN | Meningitis | Immunosupression |
| 75-2 | 1/2a | 2000 | n-MN | Meningitis | Cancer |
| 80 | 4b | 2000 | n-MN | Sepsis | Alcoholism |
| 86 | 1/2a | 2000 | n-MN | Sepsis | No indication |
| 96 | 4b | 2000 | n-MN | Sepsis | Digestive diseases |
| 97 | 1/2a | 2000 | n-MN | Unknown | Digestive diseases |
| 103 | 4b | 2000 | n-MN | Sepsis | Alcoholism |
| 107 | 1/2a | 2000 | n-MN | Sepsis | Iatrogenic immunosuppression |
| 109 | 4b | 2000 | n-MN | Meningitis | No indication |
| 111 | 1/2a | 2000 | n-MN | Meningitis + sepsis | No indication |
| 112 | 1/2a | 2000 | n-MN | Sepsis | No indication |
| 115 | 1/2b | 2000 | n-MN | Sepsis | Cancer |
| 118 | 4b | 2000 | n-MN | Sepsis | Cancer |
| 123 | 4c | 2000 | n-MN | Sepsis | Cancer |
| 126 | 1/2a | 2000 | n-MN | Sepsis | No indication |
| 128 | 1/2a | 2000 | n-MN | Meningitis + sepsis | No indication |
| 136 | 1/2a | 2000 | n-MN | Meningitis | Immunosupression |
| 144 | 4b | 2000 | n-MN | Sepsis | Digestive diseases |
| 145 | 4b | 2000 | n-MN | Unknown | Cancer |
| 148 | 1/2a | 2000 | n-MN | Sepsis | No indication |
| 149 | 4b | 2000 | n-MN | Sepsis | No indication |
| 149-2 | 4b | 2000 | n-MN | Sepsis | No indication |
| 157 | 4b | 2000 | n-MN | Sepsis | Cancer |
| 162 | 1/2a | 2000 | n-MN | Unknown | Digestive diseases |
| 167 | 4b | 2000 | n-MN | Sepsis | No indication |
| 2 | 1/2a | 2001 | n-MN | Sepsis | Respiratory diseases |
| 4 | 4b | 2001 | MN | Stillbirth | Transplantation |
| 6 | 1/2a | 2001 | n-MN | Meningitis | No indication |
| 9 | 4b | 2001 | n-MN | Meningitis | No indication |
| 10 | 4b | 2001 | n-MN | Sepsis | No indication |
| 18 | 1/2a | 2001 | n-MN | Sepsis | Cancer |
| 19 | 4b | 2001 | n-MN | Sepsis | No indication |
| 20 | 4b | 2001 | n-MN | Sepsis | Cancer |
| 24 | 4b | 2001 | n-MN | Sepsis | Iatrogenic immunosuppression |
| 28 | 1/2a | 2001 | n-MN | Sepsis | No indication |
| 32 | 4b | 2001 | n-MN | Sepsis | Cancer |
| 34 | 4b | 2001 | n-MN | Sepsis | No indication |
| 35 | 1/2a | 2001 | n-MN | Sepsis | Digestive diseases |
| 36 | 4b | 2001 | n-MN | Sepsis | No indication |
| 37 | 4b | 2001 | MN | Sepsis | No indication |
| 39-1 | 4b | 2001 | n-MN | Meningitis + sepsis | No indication |
| 39-2 | 4b | 2001 | n-MN | Sepsis | No indication |
| 40 | 4b | 2001 | n-MN | Sepsis | Transplantation |
| 41 | 4b | 2001 | MN | Sepsis | No indication |
| 48 | 1/2a | 2001 | MN | Unknown | No indication |
| 50 | 1/2a | 2001 | n-MN | Sepsis | No indication |
| 53 | 4b | 2001 | n-MN | Sepsis | Chronic kidney diseases |
| 55 | 1/2a | 2001 | n-MN | Sepsis | Immunosupression |
| 60 | 4b | 2001 | n-MN | Sepsis | Cancer |
| 61 | 4b | 2001 | n-MN | Sepsis | No indication |
| 63 | 4b | 2001 | n-MN | Sepsis | No indication |
| 65 | 1/2a | 2001 | n-MN | Sepsis | Cancer |
| 66 | 4b | 2001 | n-MN | Sepsis | Cancer |
| 67 | 4b | 2001 | n-MN | Meningitis | No indication |
| 72 | 4b | 2001 | MN | Unknown | No indication |
| 75 | 1/2a | 2001 | n-MN | Sepsis | No indication |
| 76 | 4b | 2001 | n-MN | Unknown | No indication |
| 77 | 4b | 2001 | n-MN | Sepsis | No indication |
| 78 | 1/2a | 2001 | n-MN | Meningitis + sepsis | Cancer |
| 81 | 1/2c | 2001 | n-MN | Sepsis | Respiratory diseases |
| 84 | 1/2b | 2001 | n-MN | Sepsis | Digestive diseases |
| 85 | 1/2a | 2001 | n-MN | Sepsis | Cancer |
| 91 | 1/2b | 2001 | n-MN | Sepsis | No indication |
| 95 | 4b | 2001 | n-MN | Unknown | No indication |
| 96 | 4b | 2001 | n-MN | Sepsis | Cancer |
| 102-01 | 1/2b | 2001 | n-MN | Sepsis | Digestive diseases |
| 102-02 | 1/2b | 2001 | n-MN | Sepsis | Chronic kidney diseases |
| 103 | 1/2a | 2001 | MN | Premature birth | No indication |
| 104 | 1/2a | 2001 | n-MN | Sepsis | Digestive diseases |
| 108 | 1/2a | 2001 | n-MN | Meningitis + sepsis | No indication |
| 114 | 1/2a | 2001 | MN | Meningitis | No indication |
| 121 | 4b | 2001 | n-MN | Sepsis | Digestive diseases |
| 123 | 4b | 2001 | n-MN | Sepsis | No indication |
| 124 | 1/2a | 2001 | n-MN | Sepsis | Cancer |
| 129 | 1/2a | 2001 | n-MN | Sepsis | No indication |
| 4 | 1/2a | 2002 | MN | Premature birth | No indication |
| 8 | 1/2a | 2002 | n-MN | Sepsis | Cancer |
| 20 | 4b | 2002 | MN | Unknown | No indication |
| 36 | 1/2a | 2002 | n-MN | Sepsis | No indication |
| 57 | 4a | 2002 | n-MN | Sepsis | Respiratory diseases |
| 64 | 1/2a | 2002 | n-MN | Sepsis | Cancer |
| 67 | 1/2b | 2002 | n-MN | Sepsis | Digestive diseases |
| 75 | 4b | 2002 | n-MN | Sepsis | Respiratory diseases |
| 76 | 1/2a | 2002 | n-MN | Meningitis | No indication |
| 82 | 1/2b | 2002 | n-MN | Sepsis | No indication |
| 89 | 1/2a | 2002 | n-MN | Sepsis | Respiratory diseases |
| 102 | 1/2a | 2002 | n-MN | Sepsis | Cancer |
| 108 | 1/2b | 2002 | n-MN | Sepsis | Immunosupression |
| 111 | 1/2a | 2002 | n-MN | Meningitis | No indication |
| 120 | 1/2a | 2002 | n-MN | Meningitis + sepsis | No indication |
| 121 | 1/2a | 2002 | n-MN | Sepsis | Cancer |
| 126 | 1/2a | 2002 | n-MN | Sepsis | No indication |
| 130 | 4b | 2002 | n-MN | Meningitis | No indication |
| 135 | 1/2b | 2002 | n-MN | Sepsis | No indication |
| 141 | 1/2a | 2002 | n-MN | Sepsis | Respiratory diseases |
| 145 | 4a | 2002 | n-MN | Sepsis | No indication |
| 156 | 1/2a | 2002 | n-MN | Meningitis + sepsis | No indication |
| 159 | 4b | 2002 | n-MN | Meningitis + sepsis | No indication |
| 160 | 1/2a | 2002 | MN | Premature birth | No indication |
| 162 | 1/2b | 2002 | n-MN | Sepsis | No indication |
| 166 | 1/2a | 2002 | n-MN | Sepsis | Transplantation |
| 171 | 1/2b | 2002 | n-MN | Sepsis | Digestive diseases |
| 173 | 1/2a | 2002 | n-MN | Sepsis | Cancer |
| 180 | 3a | 2002 | n-MN | Sepsis | No indication |
| 182 | 3a | 2002 | n-MN | Sepsis | Cancer |
| 184 | 1/2a | 2002 | n-MN | Sepsis | No indication |
| 185 | 3a | 2002 | n-MN | Sepsis | Cancer |
| 2 | 1/2a | 2003 | n-MN | Sepsis | Respiratory diseases |
| 6 | 4b | 2003 | n-MN | Sepsis | Respiratory diseases |
| 8 | 4b | 2003 | n-MN | Meningitis | No indication |
| 9 | 4b | 2003 | n-MN | Sepsis | Cancer |
| 12 | 1/2a | 2003 | MN | Premature birth | No indication |
| 14 | 4b | 2003 | n-MN | Meningitis + sepsis | Cancer |
| 16 | 1/2a | 2003 | n-MN | Sepsis | No indication |
| 17 | 1/2b | 2003 | n-MN | Sepsis | Cancer |
| 21 | 1/2a | 2003 | n-MN | Sepsis | Cancer |
| 23 | 1/2b | 2003 | n-MN | Sepsis | Chronic kidney diseases |
| 29 | 1/2b | 2003 | n-MN | Sepsis | No indication |
| 31 | 4b | 2003 | n-MN | Sepsis | Chronic kidney diseases |
| 42 | 4b | 2003 | n-MN | Sepsis | Cancer |
| 51 | 1/2b | 2003 | n-MN | Sepsis | No indication |
| 55 | 4b | 2003 | n-MN | Sepsis | Cancer |
| 56 | 3a | 2003 | n-MN | Sepsis | Alcoholism |
| 57 | 4b | 2003 | n-MN | Sepsis | Cancer |
| 60 | 1/2b | 2003 | n-MN | Sepsis | Heart diseases |
| 61 | 1/2b | 2003 | n-MN | Sepsis | Heart diseases |
| 73 | 1/2a | 2003 | n-MN | Sepsis | Respiratory diseases |
| 74 | 4b | 2003 | n-MN | Meningitis + sepsis | Transplantation |
| 75 | 4b | 2003 | n-MN | Sepsis | Cancer |
| 78 | 4b | 2003 | MN | Premature birth | No indication |
| 79 | 4b | 2003 | n-MN | Sepsis | Cancer |
| 80 | 1/2a | 2003 | n-MN | Sepsis | Digestive diseases |
| 89 | 1/2a | 2003 | n-MN | Peritonitis | Transplantation |
| 90 | 4b | 2003 | n-MN | Sepsis | Cancer |
| 95 | 1/2b | 2003 | n-MN | Sepsis | No indication |
| 97 | 1/2b | 2003 | MN | Stillbirth | No indication |
| 99 | 1/2a | 2003 | n-MN | Sepsis | Cancer |
| 100 | 4b | 2003 | n-MN | Sepsis | No indication |
| 101 | 1/2a | 2003 | n-MN | Sepsis | Chronic kidney diseases |
| 103 | 4b | 2003 | n-MN | Sepsis | Cancer |
| 107 | 4b | 2003 | n-MN | Unknown | No indication |
| 112 | 1/2a | 2003 | n-MN | Sepsis | No indication |
| 113 | 1/2a | 2003 | n-MN | Sepsis | Autoimmune diseases |
| 115 | 1/2a | 2003 | n-MN | Sepsis | Cancer |
| 116 | 1/2a | 2003 | n-MN | Sepsis | No indication |
| 117 | 1/2a | 2003 | n-MN | Meningitis + sepsis | No indication |
| 118 | 4b | 2003 | n-MN | Sepsis | No indication |
| 122 | 1/2a | 2003 | n-MN | Sepsis | Transplantation |
| 127 | 1/2a | 2003 | n-MN | Meningitis + sepsis | Chronic kidney diseases |
| 132 | 1/2a | 2003 | n-MN | Sepsis | Cancer |
| 134 | 1/2a | 2003 | n-MN | Sepsis | No indication |
| 137 | 1/2a | 2003 | n-MN | Sepsis | Digestive diseases |
| 138 | 4b | 2003 | n-MN | Sepsis | Immunosupression |
| 141 | 4b | 2003 | MN | Stillbirth | No indication |
| 144 | 4b | 2003 | n-MN | Sepsis | No indication |
| 147 | 4b | 2003 | MN | Sepsis | No indication |
| 148 | 1/2a | 2003 | n-MN | Sepsis | Cancer |
| 149 | 4b | 2003 | n-MN | Meningitis | No indication |
| 150 | 1/2a | 2003 | n-MN | Sepsis | Heart diseases |
| 151 | 4b | 2003 | MN | Sepsis | No indication |
| 160 | 4b | 2003 | n-MN | Sepsis | No indication |
| 161 | 1/2b | 2003 | MN | Stillbirth | No indication |
| 163 | 4b | 2003 | n-MN | Sepsis | Cancer |
| 166 | 1/2a | 2003 | n-MN | Sepsis | No indication |
| 169 | 4b | 2003 | n-MN | Meningitis | Cancer |
| 172 | 1/2a | 2003 | n-MN | Sepsis | Cancer |
| 173 | 1/2b | 2003 | n-MN | Sepsis | No indication |
| 174 | 1/2b | 2003 | n-MN | Sepsis | No indication |
| 179 | 1/2a | 2003 | n-MN | Sepsis | Chronic kidney diseases |
| 190/1 | 4b | 2003 | n-MN | Sepsis | No indication |
| 190/2 | 1/2a | 2003 | MN | Premature birth | No indication |
| 1 | 4b | 2004 | n-MN | Sepsis | Cancer |
| 2 | 1/2b | 2004 | n-MN | Sepsis | Digestive diseases |
| 3 | 1/2a | 2004 | n-MN | Sepsis | Cancer |
| 4 | 4b | 2004 | n-MN | Sepsis | No indication |
| 5 | 1/2a | 2004 | n-MN | Sepsis | No indication |
| 6 | 1/2a | 2004 | n-MN | Sepsis | No indication |
| 7 | 1/2a | 2004 | n-MN | Sepsis | Cancer |
| 13 | 1/2a | 2004 | n-MN | Meningitis | No indication |
| 16 | 1/2a | 2004 | n-MN | Sepsis | No indication |
| 19 | 1/2a | 2004 | n-MN | Sepsis | Cancer |
| 21 | 1/2b | 2004 | n-MN | Sepsis | No indication |
| 23 | 4b | 2004 | n-MN | Meningitis | Cancer |
| 25 | 4b | 2004 | MN | Sepsis | No indication |
| 38 | 1/2a | 2004 | n-MN | Sepsis | Cancer |
| 63 | 4b | 2004 | n-MN | Sepsis | Cancer |
| 68 | 1/2a | 2004 | MN | Premature birth | No indication |
| 82 | 1/2a | 2004 | n-MN | Sepsis | No indication |
| 100 | 1/2b | 2004 | n-MN | Sepsis | No indication |
| 150 | 4b | 2004 | MN | Stillbirth | No indication |
| 164 | 1/2a | 2004 | n-MN | Sepsis | Cancer |
| 171 | 4b | 2004 | n-MN | Meningitis + sepsis | No indication |
| 180 | 1/2c | 2004 | n-MN | Sepsis | Cancer |
| 185 | 4b | 2004 | n-MN | Meningitis + sepsis | No indication |
| 186 | 1/2b | 2004 | n-MN | Sepsis | No indication |
| 187 | 1/2a | 2004 | n-MN | Sepsis | No indication |
| 188 | 1/2a | 2004 | n-MN | Peritonitis | Digestive diseases |
| 194 | 1/2a | 2004 | n-MN | Sepsis | Immunosupression |
| 195 | 1/2a | 2004 | n-MN | Unknown | No indication |
| 197 | 1/2a | 2004 | n-MN | Sepsis | Cancer |
| 202 | 1/2a | 2004 | n-MN | Sepsis | No indication |
| 207 | 1/2b | 2004 | n-MN | Sepsis | No indication |
| 211 | 1/2a | 2004 | n-MN | Sepsis | Digestive diseases |
| 219 | 1/2a | 2004 | n-MN | Sepsis | Cancer |
| 222 | 1/2a | 2004 | n-MN | Sepsis | No indication |
| 227 | 4b | 2004 | MN | Sepsis | No indication |
| 229 | 4b | 2004 | MN | Premature birth | No indication |
| 231 | 1/2a | 2004 | n-MN | Sepsis | Heart diseases |
| 233 | 4b | 2004 | n-MN | Meningitis + sepsis | No indication |
| 241 | 1/2b | 2004 | n-MN | Sepsis | No indication |
| 245 | 1/2b | 2004 | n-MN | Sepsis | Cancer |
| 252 | 1/2a | 2004 | n-MN | Sepsis | Chronic kidney diseases |
| 254 | 1/2a | 2004 | n-MN | Sepsis | Cancer |
| 255 | 4b | 2004 | n-MN | Sepsis | Respiratory diseases |
| 259 | 4b | 2004 | n-MN | Meningitis + sepsis | No indication |
| 270 | 4b | 2004 | n-MN | Sepsis | Heart diseases |
| 271 | 1/2b | 2004 | n-MN | Sepsis | No indication |
| 283 | 1/2a | 2004 | n-MN | Meningitis | No indication |
| 289 | 1/2a | 2004 | n-MN | Sepsis | Iatrogenic immunosuppression |
| 293 | 4b | 2004 | n-MN | Meningitis | Iatrogenic immunosuppression |
| 297 | 1/2a | 2004 | MN | Sepsis | No indication |
| 304 | 4b | 2004 | n-MN | Meningitis + sepsis | No indication |
| 305 | 1/2a | 2004 | n-MN | Sepsis | No indication |
| 306 | 1/2b | 2004 | MN | Stillbirth | No indication |
| 307 | 1/2b | 2004 | n-MN | Sepsis | No indication |
| 308 | 4b | 2004 | n-MN | Sepsis | No indication |
| 314 | 1/2a | 2004 | n-MN | Sepsis | Iatrogenic immunosuppression |
| 326 | 1/2a | 2004 | MN | Premature birth | No indication |
| 328 | 4b | 2004 | MN | Unknown | No indication |
| 334 | 4b | 2004 | n-MN | Sepsis | No indication |
| 337 | 4b | 2004 | n-MN | Sepsis | Heart diseases |
| 338 | 1/2b | 2004 | n-MN | Sepsis | Immunosupression |
| 344 | 4b | 2004 | n-MN | Sepsis | Transplantation |
| 346 | 1/2c | 2004 | n-MN | Sepsis | Cancer |
| 348 | 1/2c | 2004 | n-MN | Sepsis | Immunosupression |
| 356 | 1/2a | 2004 | n-MN | Sepsis | No indication |
| 357 | 4b | 2004 | n-MN | Sepsis | No indication |
| 363 | 1/2a | 2004 | n-MN | Sepsis | Immunosupression |
| 367 | 1/2a | 2004 | n-MN | Sepsis | Digestive diseases |
| 1 | 4b | 2005 | n-MN | Meningitis + sepsis | Transplantation |
| 12 | 1/2a | 2005 | n-MN | Sepsis | Immunosupression |
| 13 | 1/2a | 2005 | n-MN | Sepsis | No indication |
| 14 | 1/2b | 2005 | n-MN | Sepsis | Chronic kidney diseases |
| 16 | 1/2a | 2005 | n-MN | Sepsis | Alcoholism |
| 20 | 1/2a | 2005 | n-MN | Sepsis | Respiratory diseases |
| 23 | 1/2a | 2005 | n-MN | Sepsis | No indication |
| 34 | 1/2a | 2005 | n-MN | Sepsis | No indication |
| 38 | 4b | 2005 | n-MN | Sepsis | No indication |
| 45 | 4b | 2005 | n-MN | Sepsis | No indication |
| 57 | 4b | 2005 | MN | Stillbirth | No indication |
| 58 | 4b | 2005 | n-MN | Sepsis | Respiratory diseases |
| 62 | 1/2a | 2005 | n-MN | Sepsis | No indication |
| 66 | 4b | 2005 | n-MN | Unknown | No indication |
| 73 | 4b | 2005 | n-MN | Sepsis | Cancer |
| 79 | 4b | 2005 | n-MN | Sepsis | No indication |
| 90 | 1/2b | 2005 | n-MN | Sepsis | Heart diseases |
| 91 | 1/2c | 2005 | n-MN | Sepsis | Cancer |
| 112 | 4b | 2005 | n-MN | Sepsis | No indication |
| 117 | 1/2a | 2005 | n-MN | Sepsis | No indication |
| 127 | 4b | 2005 | n-MN | Sepsis | No indication |
| 134 | 1/2a | 2005 | n-MN | Sepsis | Cancer |
| 137 | 4b | 2005 | n-MN | Sepsis | No indication |
| 142 | 4b | 2005 | n-MN | Sepsis | Alcoholism |
| 144 | 1/2a | 2005 | n-MN | Sepsis | No indication |
| 166 | 4b | 2005 | n-MN | Sepsis | Digestive diseases |
| 167 | 4b | 2005 | n-MN | Sepsis | Autoimmune diseases |
| 175 | 4b | 2005 | MN | Sepsis | No indication |
| 189 | 1/2b | 2005 | n-MN | Meningitis + sepsis | Digestive diseases |
| 193 | 1/2a | 2005 | n-MN | Sepsis | No indication |
| 177 | 1/2b | 2005 | n-MN | Sepsis | Cancer |
| 178 | 1/2a | 2005 | n-MN | Meningitis + sepsis | Alcoholism |
| 198 | 1/2b | 2005 | n-MN | Sepsis | No indication |
| 210 | 4b | 2005 | n-MN | Sepsis | No indication |
| 213 | 1/2a | 2005 | n-MN | Sepsis | No indication |
| 216 | auto | 2005 | n-MN | Sepsis | No indication |
| 218 | 1/2a | 2005 | n-MN | Sepsis | Immunosupression |
| 223 | 1/2a | 2005 | n-MN | Meningitis + sepsis | Digestive diseases |
| 237 | 1/2a | 2005 | n-MN | Meningitis | Respiratory diseases |
| 238 | 4b | 2005 | n-MN | Meningitis + sepsis | Respiratory diseases |
| 1 | 1/2a | 2006 | n-MN | Unknown | Surgery |
| 3 | 1/2a | 2006 | n-MN | Unknown | Immunosupression |
| 5 | 1/2a | 2006 | n-MN | Sepsis | No indication |
| 17 | 1/2c | 2006 | n-MN | Sepsis | Cancer |
| 22 | 4b | 2006 | n-MN | Sepsis | No indication |
| 29 | 4b | 2006 | n-MN | Sepsis | Digestive diseases |
| 30 | 1/2b | 2006 | n-MN | Sepsis | Cancer |
| 50 | 1/2a | 2006 | n-MN | Sepsis | No indication |
| 56 | 4b | 2006 | n-MN | Sepsis | Respiratory diseases |
| 67 | 1/2a | 2006 | n-MN | Sepsis | Cancer |
| 78 | 1/2a | 2006 | n-MN | Sepsis | Cancer |
| 86 | 1/2a | 2006 | n-MN | Sepsis | Heart diseases |
| 87 | 1/2a | 2006 | n-MN | Sepsis | No indication |
| 110 | 4b | 2006 | n-MN | Sepsis | No indication |
| 111 | 1/2b | 2006 | n-MN | Sepsis | Cancer |
| 128 | 1/2b | 2006 | n-MN | Sepsis | Immunosupression |
| 146 | 1/2b | 2006 | n-MN | Sepsis | No indication |
| 153 | 1/2a | 2006 | n-MN | Sepsis | Alcoholism |
| 154 | 1/2a | 2006 | n-MN | Sepsis | No indication |
| 174 | 1/2a | 2006 | n-MN | Sepsis | Cancer |
| 191 | 4b | 2006 | MN | Gastro-enteritis | No indication |
| 192 | 1/2a | 2006 | n-MN | Sepsis | Cancer |
| 194 | 1/2b | 2006 | n-MN | Sepsis | Cancer |
| 195 | 1/2a | 2006 | n-MN | Sepsis | No indication |
| 198 | 1/2a | 2006 | n-MN | Meningitis + sepsis | No indication |
| 200 | 1/2b | 2006 | n-MN | Sepsis | Heart diseases |
| 201 | 1/2b | 2006 | n-MN | Sepsis | No indication |
| 203 | 1/2a | 2006 | n-MN | Sepsis | Cancer |
| 205 | 1/2a | 2006 | n-MN | Sepsis | Cancer |
| 213 | 1/2a | 2006 | n-MN | Sepsis | Respiratory diseases |
| 220 | 1/2a | 2006 | n-MN | Sepsis | No indication |
| 231 | 1/2a | 2006 | n-MN | Sepsis | No indication |
| 235 | 4b | 2006 | MN | Sepsis | No indication |
| 244 | 4b | 2006 | n-MN | Sepsis | No indication |
| 249 | 4b | 2006 | n-MN | Meningitis + sepsis | No indication |
| 258 | 1/2a | 2006 | n-MN | Sepsis | No indication |
| 259 | 4b | 2006 | n-MN | Sepsis | No indication |
| 270 | 4b | 2006 | n-MN | Sepsis | No indication |
| 271 | 1/2a | 2006 | n-MN | Sepsis | Cancer |
| 282 | 4b | 2006 | n-MN | Meningitis | Heart diseases |
| 283 | 1/2a | 2006 | n-MN | Meningitis | No indication |
| 293 | 4b | 2006 | n-MN | Meningitis | Immunosupression |
| 297 | 4b | 2006 | n-MN | Meningitis | No indication |
| 304 | 4b | 2006 | n-MN | Sepsis | Chronic kidney diseases |
| 305 | 4b | 2006 | n-MN | Sepsis | Heart diseases |
| 307 | 1/2a | 2006 | n-MN | Sepsis | No indication |
| 313 | 1/2a | 2006 | n-MN | Sepsis | Immunosupression |
| 321 | 4b | 2006 | n-MN | Sepsis | No indication |
| 322 | 4b | 2006 | MN | Sepsis | No indication |
| 327 | 1/2a | 2006 | n-MN | Sepsis | Chronic kidney diseases |
| 328 | 1/2a | 2006 | n-MN | Meningitis + sepsis | Immunosupression |
| 340 | 4b | 2006 | n-MN | Sepsis | Cancer |
| 341 | 1/2a | 2006 | n-MN | Unknown | No indication |
| 344 | 1/2a | 2006 | MN | Stillbirth | No indication |
| 371 | 4b | 2006 | n-MN | Sepsis | No indication |
| 07-10 | 4b | 2007 | n-MN | Sepsis | No indication |
| 07-14 | 1/2a | 2007 | n-MN | Sepsis | Cancer |
| 07-17 | 4b | 2007 | n-MN | Sepsis | Cancer |
| 07-18 | 4b | 2007 | MN | Sepsis | No indication |
| 07-24 | 4b | 2007 | n-MN | Sepsis | Chronic kidney diseases |
| 07-25 | 1/2a | 2007 | MN | Sepsis | No indication |
| 07-34 | 4b | 2007 | MN | Premature birth | No indication |
| 07-35 | 4b | 2007 | MN | Sepsis | No indication |
| 07-26 | 4b | 2007 | n-MN | Sepsis | Cancer |
| 07-36 | 4b | 2007 | n-MN | Sepsis | No indication |
| 07-42 | 1/2b | 2007 | n-MN | pustulosis | No indication |
| 07-47 | 4b | 2007 | n-MN | Meningitis + sepsis | No indication |
| 07-53 | 1/2a | 2007 | n-MN | Sepsis | Chronic kidney diseases |
| 07-55 | 1/2a | 2007 | n-MN | Sepsis | Iatrogenic immunosuppression |
| 07-56 | 4b | 2007 | n-MN | Sepsis | No indication |
| 07-63 | 4b | 2007 | n-MN | Sepsis | No indication |
| 07-64 | 1/2c | 2007 | n-MN | Sepsis | Cancer |
| 07-73 | 4b | 2007 | n-MN | Sepsis | No indication |
| 07-78 | 1/2a | 2007 | n-MN | Sepsis | No indication |
| 07-81 | 4b | 2007 | n-MN | Sepsis | Chronic kidney diseases |
| 07-98 | 4b | 2007 | n-MN | Sepsis | Cancer |
| 07-99 | 4b | 2007 | n-MN | Sepsis | No indication |
| 07-102 | 1/2a | 2007 | MN | Unknown | No indication |
| 07-108 | 4b | 2007 | n-MN | Sepsis | Cancer |
| 07-109 | 1/2a | 2007 | n-MN | Sepsis | Cancer |
| 07-115 | 1/2a | 2007 | n-MN | Sepsis | Cancer |
| 07-121 | 4b | 2007 | n-MN | Meningitis | No indication |
| 07-122 | 4b | 2007 | n-MN | Sepsis | No indication |
| 07-126 | 4b | 2007 | n-MN | Sepsis | Cancer |
| 07-127 | 1/2a | 2007 | n-MN | Sepsis | No indication |
| 07-130 | 4b | 2007 | n-MN | Sepsis | No indication |
| 07-138 | 1/2a | 2007 | n-MN | Sepsis | No indication |
| 07-140 | 1/2a | 2007 | n-MN | Sepsis | No indication |
| 07-141 | 4b | 2007 | n-MN | Sepsis | No indication |
| 07-145 | 4b | 2007 | n-MN | Sepsis | Respiratory diseases |
| 07-146 | 4b | 2007 | n-MN | Sepsis | No indication |
| 07-150 | 4b | 2007 | MN | Unknown | No indication |
| 07-152 | 1/2a | 2007 | n-MN | Sepsis | No indication |
| 07-155 | 1/2a | 2007 | n-MN | Sepsis | Cancer |
| 07-156 | 4b | 2007 | n-MN | Sepsis | No indication |
| 07-157 | 4b | 2007 | n-MN | Meningitis | No indication |
| 07-161 | 4e | 2007 | n-MN | Meningitis + sepsis | Cancer |
| 07-163 | 4b | 2007 | n-MN | Sepsis | No indication |
| 07-167 | 1/2a | 2007 | n-MN | Sepsis | No indication |
| 07-169 | 4b | 2007 | n-MN | Sepsis | Chronic kidney diseases |
| 07-171 | 1/2a | 2007 | n-MN | Sepsis | No indication |
| 07-174 | 4b | 2007 | n-MN | Sepsis | Cancer |
| 07-175 | 4b | 2007 | n-MN | Unknown | Surgery |
| 07-181 | 4b | 2007 | n-MN | Sepsis | Iatrogenic immunosuppression |
| 07-182 | 1/2b | 2007 | n-MN | Sepsis | Cancer |
| 07-189 | 4b | 2007 | n-MN | Meningitis | No indication |
| 07-202 | 4b | 2007 | n-MN | Meningitis + sepsis | Cancer |
| 08-03 | 4e | 2008 | n-MN | Sepsis | No indication |
| 08-09 | 1/2a | 2008 | n-MN | Meningitis | No indication |
| 08-17 | 1/2b | 2008 | n-MN | Sepsis | No indication |
| 08-18 | 4b | 2008 | n-MN | Sepsis | No indication |
| 08-21 | 4b | 2008 | MN | Premature birth | |
| 08-23 | 4b | 2008 | MN | Sepsis |  |
| 08-25 | 4b | 2008 | n-MN | Meningitis | No indication |
| 08-26 | 1/2c | 2008 | n-MN | Sepsis | Transplantation |
| 08-29 | 1/2a | 2008 | n-MN | Sepsis | Cancer |
| 08-31 | 1/2a | 2008 | n-MN | pustulosis | No indication |
| 08-32 | 1/2b | 2008 | n-MN | Sepsis | Transplantation |
| 08-34 | 4b | 2008 | n-MN | Sepsis | No indication |
| 08-36 | 1/2a | 2008 | n-MN | Sepsis | Transplantation |
| 08-37 | 1/2b | 2008 | MN | Sepsis | No indication |
| 08-38 | 4b | 2008 | n-MN | Sepsis | Digestive diseases |
| 08-39 | 1/2a | 2008 | n-MN | Meningitis + sepsis | Iatrogenic immunosuppression |
| 08-40 | 1/2a | 2008 | n-MN | Sepsis | Cancer |
| 08-43 | 4b | 2008 | n-MN | Sepsis | No indication |
| 08-44 | 1/2a | 2008 | n-MN | Meningitis | Respiratory diseases |
| 08-46 | 1/2b | 2008 | n-MN | Meningitis + sepsis | No indication |
| 08-51 | 1/2b | 2008 | n-MN | Peritonitis | Digestive diseases |
| 08-54 | 1/2b | 2008 | n-MN | Sepsis | No indication |
| 08-64 | 1/2a | 2008 | n-MN | Sepsis | Cancer |
| 08-68 | 4b | 2008 | n-MN | Meningitis + sepsis | Iatrogenic immunosuppression |
| 08-70 | 1/2a | 2008 | n-MN | Sepsis | Cancer |
| 08-73 | 1/2b | 2008 | n-MN | Sepsis | Surgery |
| 08-74 | 1/2a | 2008 | n-MN | Unknown | No indication |
| 08-77 | 1/2a | 2008 | n-MN | Sepsis | Surgery |
| 08-78 | 4b | 2008 | n-MN | Sepsis | Immunosupression |
| .08-81 | 4b | 2008 | n-MN | Meningitis + sepsis | Immunosupression |
| 08-83 | 1/2a | 2008 | n-MN | Sepsis | No indication |
| 08-85 | 1/2a | 2008 | n-MN | Sepsis | Cancer |
| 08-86 | 4b | 2008 | n-MN | Sepsis | No indication |
| 08-90 | 4b | 2008 | MN | Premature birth | No indication |
| 08-101 | 4b | 2008 | n-MN | Sepsis | Chronic kidney diseases |
| 08-113 | 1/2b | 2008 | n-MN | Sepsis | No indication |
| 08-114 | sg4 | 2008 | n-MN | Meningitis | Immunosupression |
| 08-128 | 1/2a | 2008 | n-MN | Sepsis | Cancer |
| 08-130 | 4b | 2008 | n-MN | Sepsis | Immunosupression |
| 08-144 | 1/2a | 2008 | n-MN | Sepsis | Chronic kidney diseases |
| 08-143 | 1/2a | 2008 | n-MN | Sepsis | No indication |
| 08-149 | 1/2b | 2008 | n-MN | Sepsis | Alcoholism |
| 08-158 | 4b | 2008 | n-MN | Sepsis | Cancer |
| 08-186 | 1/2a | 2008 | MN | Sepsis | No indication |
| 08-201 | 1/2b | 2008 | n-MN | Sepsis | Cancer |
| 08-202 | 1/2a | 2008 | n-MN | Sepsis | Chronic kidney diseases |
| 08-203 | 1/2a | 2008 | n-MN | Sepsis | Chronic kidney diseases |
| 08-205 | 1/2a | 2008 | n-MN | Sepsis | Cancer |
| 08-209 | 1/2a | 2008 | n-MN | Sepsis | Cancer |
| 08-210 | 4b | 2008 | n-MN | Sepsis | No indication |
| 08-242 | 4b | 2008 | n-MN | Gastro-enteritis | Digestive diseases |
| 08-244 | 4b | 2008 | n-MN | Sepsis | No indication |
| 08-246 | 1/2a | 2008 | n-MN | Sepsis | Immunosupression |
| 09-001 | 1/2b | 2009 | n-MN | Sepsis | Chronic kidney diseases |
| 09-002 | 1/2a | 2009 | n-MN | Sepsis | Immunosupression |
| 09-011 | 1/2a | 2009 | n-MN | Sepsis | No indication |
| 09-012 | 1/2a | 2009 | n-MN | Sepsis | No indication |
| 09-013 | 1/2a | 2009 | n-MN | Sepsis | Cancer |
| 09-014 | 1/2a | 2009 | n-MN | Sepsis | No indication |
| 09-016 | 1/2a | 2009 | n-MN | Sepsis | No indication |
| 09-017 | 1/2a | 2009 | n-MN | Sepsis | Chronic kidney diseases |
| 09-024 | 1/2a | 2009 | n-MN | Sepsis | No indication |
| 09-030 | 4b | 2009 | n-MN | Sepsis | No indication |
| 09-031 | 4b | 2009 | n-MN | Sepsis | Cancer |
| 09-033 | 4b | 2009 | n-MN | Unknown | No indication |
| 09-039 | 4b | 2009 | MN | Other | No indication |
| 09-040 | 1/2a | 2009 | n-MN | Sepsis | No indication |
| 09-041 | 4b | 2009 | n-MN | Meningitis + sepsis | No indication |
| 09-051 | 1/2a | 2009 | n-MN | Sepsis | No indication |
| 09-057 | 1/2a | 2009 | n-MN | Sepsis | Cancer |
| 09-064 | 4b | 2009 | n-MN | Unknown | Surgery |
| 09-072 | 1/2a | 2009 | n-MN | Sepsis | Cancer |
| 09-073 | 1/2a | 2009 | n-MN | Sepsis | No indication |
| 09-075 | 1/2a | 2009 | n-MN | Sepsis | No indication |
| 09-076 | 1/2a | 2009 | n-MN | Sepsis | Iatrogenic immunosuppression |
| 09-077 | 4b | 2009 | n-MN | Unknown | Surgery |
| 09-080 | 4b | 2009 | n-MN | Sepsis | No indication |
| 09-083 | 1/2a | 2009 | n-MN | Sepsis | Cancer |
| 09-084 | 1/2a | 2009 | n-MN | Sepsis | No indication |
| 09-101 | 1/2a | 2009 | n-MN | Sepsis | No indication |
| 09-103 | 1/2b | 2009 | n-MN | Sepsis | Cancer |
| 09-129 | 1/2a | 2009 | n-MN | Sepsis | Cancer |
| 09-133 | 4b | 2009 | n-MN | Sepsis | Iatrogenic immunosuppression |
| 09-134 | 1/2a | 2009 | n-MN | Sepsis | No indication |
| 09-136 | 1/2b | 2009 | n-MN | Sepsis | No indication |
| 09-145 | 4b | 2009 | n-MN | Sepsis | Cancer |
| 09-146 | 1/2a | 2009 | n-MN | Unknown | No indication |
| 09-147 | 4b | 2009 | MN | Meningitis | No indication |
| 09-165 | 1/2b | 2009 | n-MN | Sepsis | Transplantation |
| 09-173 | 1/2a | 2009 | n-MN | Sepsis | No indication |
| 09-174 | 1/2a | 2009 | n-MN | Sepsis | Immunosupression |
| 09-175 | 1/2a | 2009 | n-MN | Meningitis + sepsis | Digestive diseases |
| 09-177 | 1/2b | 2009 | n-MN | Meningitis + sepsis | No indication |
| 09-132 | 1/2a | 2009 | n-MN | Sepsis | No indication |
| 09-179 | 4b | 2009 | n-MN | Meningitis + sepsis | Immunosupression |
| 09-182 | 1/2a | 2009 | n-MN | Sepsis | No indication |
| 09-185 | 1/2a | 2009 | n-MN | Sepsis | No indication |
| 09-186 | 1/2a | 2009 | n-MN | Sepsis | Chronic kidney diseases |
| 09-193 | 1/2a | 2009 | n-MN | Meningitis + sepsis | No indication |
| 09-196 | 4b | 2009 | n-MN | Sepsis | No indication |
| 09-197 | 4b | 2009 | n-MN | Sepsis | Iatrogenic immunosuppression |
| 09-198 | 1/2a | 2009 | n-MN | Sepsis | No indication |
| 09-218 | 1/2a | 2009 | n-MN | Sepsis | No indication |
| 09-219 | 1/2a | 2009 | n-MN | Sepsis | No indication |
| 09-229 | 1/2a | 2009 | n-MN | Sepsis | No indication |
| 09-237 | 4b | 2009 | n-MN | Sepsis | Digestive diseases |
| 09-238 | 1/2a | 2009 | n-MN | Meningitis + sepsis | No indication |
| 09-241 | 1/2b | 2009 | n-MN | Sepsis | No indication |
| 09-245 | 1/2a | 2009 | n-MN | Meningitis + sepsis | No indication |
| 09-251 | 1/2c | 2009 | n-MN | Sepsis | No indication |
| 09-262 | 1/2a | 2009 | n-MN | Sepsis | Cancer |
| 09-263 | 4b | 2009 | n-MN | Sepsis | No indication |
| 09-269 | 1/2a | 2009 | n-MN | Sepsis | Heart diseases |
| 09-280 | 1/2a | 2009 | n-MN | Sepsis | No indication |
| 09/287 | 1/2a | 2009 | n-MN | Sepsis | Cancer |
| 09/192 | 1/2a | 2009 | n-MN | Sepsis | Digestive diseases |
| 09/270 | 4b | 2009 | n-MN | Sepsis | No indication |
| 10/001 | 4b | 2010 | n-MN | Sepsis | Chronic kidney diseases |
| 10/012 | 4b | 2010 | n-MN | Sepsis | No indication |
| 10/035 | 4e | 2010 | n-MN | Sepsis | Respiratory diseases |
| 10/046 | 1/2a | 2010 | n-MN | Sepsis | Heart diseases |
| 10/047 | 4b | 2010 | n-MN | Sepsis | No indication |
| 10/054 | 1/2a | 2010 | n-MN | Sepsis | No indication |
| 10/056 | 4b | 2010 | n-MN | Unknown | Iatrogenic immunosuppression |
| 10/068 | 1/2b | 2010 | n-MN | Sepsis | Cancer |
| 10/098 | 1/2a | 2010 | n-MN | Sepsis | Digestive diseases |
| 10/099 | 4b | 2010 | n-MN | Sepsis | No indication |
| 10/100 | 1/2a | 2010 | n-MN | Sepsis | Cancer |
| 10/102 | 1/2a | 2010 | MN | Unknown | No indication |
| 10/110 | 4b | 2010 | n-MN | Sepsis | No indication |
| 10/121 | 1/2b | 2010 | n-MN | Sepsis | Digestive diseases |
| 10/139 | 1/2a | 2010 | n-MN | Peritonitis | Alcoholism |
| 10/144 | 1/2a | 2010 | n-MN | Unknown | infection/wound |
| 10/147 | 1/2a | 2010 | n-MN | Sepsis | Digestive diseases |
| 10/171 | 4b | 2010 | n-MN | Sepsis | Digestive diseases |
| 10/190 | 1/2b | 2010 | n-MN | Sepsis | Heart diseases |
| 10/191 | 4b | 2010 | n-MN | Sepsis | Iatrogenic immunosuppression |
| 10/192 | 1/2c | 2010 | n-MN | Sepsis | Cancer |
| 10/196 | 4b | 2010 | n-MN | Unknown | No indication |
| 10/198 | 1/2a | 2010 | n-MN | Sepsis | No indication |
| 10/204 | 4b | 2010 | n-MN | Sepsis | No indication |
| 10/206 | 1/2b | 2010 | n-MN | Sepsis | Digestive diseases |
| 10/215 | 4b | 2010 | n-MN | Sepsis | Digestive diseases |
| 10/217 | 4b | 2010 | n-MN | Sepsis | No indication |
| 10/232 | 1/2a | 2010 | n-MN | Sepsis | Chronic kidney diseases |
| 10/233 | 4b | 2010 | MN | Sepsis | No indication |
| 10/238 | 1/2a | 2010 | n-MN | Sepsis | Respiratory diseases |
| 10/239 | 4b | 2010 | MN | Sepsis | No indication |
| 10/256 | 1/2a | 2010 | n-MN | Sepsis | Digestive diseases |
| 10/269 | 4b | 2010 | MN | Sepsis | No indication |
| 10/297 | 1/2a | 2010 | n-MN | Sepsis | Heart diseases |
| 10/300 | 1/2a | 2010 | n-MN | Sepsis | No indication |
| 10/302 | 1/2a | 2010 | n-MN | Sepsis | Cancer |
| 10/303 | 4b | 2010 | n-MN | Sepsis | No indication |
| 10/309 | 4b | 2010 | n-MN | Meningitis | Iatrogenic immunosuppression |
| 10/310 | 1/2b | 2010 | MN | Sepsis | No indication |
| 10/312 | 1/2a | 2010 | MN | Stillbirth | No indication |
| 10/313 | 4b | 2010 | n-MN | Sepsis | No indication |
| 10/317 | 4b | 2010 | n-MN | Sepsis | Alcoholism |
| 10/318 | 4b | 2010 | n-MN | Sepsis | No indication |
| 10/332 | 1/2b | 2010 | n-MN | Sepsis | Cancer |
| 11/007 | 1/2a | 2011 | n-MN | Meningitis | Iatrogenic immunosuppression |
| 11/015 | 1/2a | 2011 | n-MN | Sepsis | Respiratory diseases |
| 11/018 | 1/2a | 2011 | n-MN | Peritonitis | No indication |
| 11/019 | 4b | 2011 | n-MN | Meningitis + sepsis | No indication |
| 11/021 | 1/2c | 2011 | n-MN | Sepsis | Cancer |
| 11/044 | 1/2a | 2011 | n-MN | Sepsis | No indication |
| 11/047 | 1/2a | 2011 | n-MN | Sepsis | Respiratory diseases |
| 11/054 | 1/2a | 2011 | n-MN | Sepsis | Alcoholism |
| 11/056 | 1/2c | 2011 | n-MN | Sepsis | Cancer |
| 11/057 | 1/2a | 2011 | n-MN | Sepsis | Cancer |
| 11/060 | 1/2a | 2011 | n-MN | Sepsis | Cancer |
| 11/075 | 4b | 2011 | MN | Sepsis | No indication |
| 11/077 | 1/2a | 2011 | n-MN | Sepsis | Respiratory diseases |
| 11/083 | 4b | 2011 | n-MN | Sepsis | Cancer |
| 11/084 | 4b | 2011 | n-MN | Meningitis | No indication |
| 11/085 | 4b | 2011 | n-MN | Sepsis | No indication |
| 11/086 | 4b | 2011 | n-MN | Sepsis | Cancer |
| 11/087 | 1/2a | 2011 | n-MN | Sepsis | No indication |
| 11/088 | 1/2a | 2011 | n-MN | Sepsis | Cancer |
| 11/093 | 1/2a | 2011 | n-MN | Sepsis | No indication |
| 11/100 | 1/2a | 2011 | n-MN | Sepsis | No indication |
| 11/104 | 4b | 2011 | n-MN | Unknown | No indication |
| 11/107 | 1/2a | 2011 | n-MN | Sepsis | No indication |
| 11/108 | 1/2a | 2011 | n-MN | Sepsis | Alcoholism |
| 11/089 | 1/2a | 2011 | n-MN | Sepsis | No indication |
| 11/090 | 1/2a | 2011 | n-MN | Sepsis | No indication |
| 11/102 | 1/2a | 2011 | n-MN | Sepsis | No indication |
| 11/113 | 4b | 2011 | n-MN | Sepsis | No indication |
| 11/114 | 1/2b | 2011 | n-MN | Sepsis | No indication |
| 11/122 | 1/2b | 2011 | n-MN | Sepsis | Cancer |
| 11/124 | 1/2a | 2011 | n-MN | Sepsis | No indication |
| 11/131 | 1/2a | 2011 | n-MN | Unknown | Cancer |
| 11/132 | 1/2a | 2011 | n-MN | Sepsis | Chronic kidney diseases |
| 11/136 | 1/2b | 2011 | n-MN | Sepsis | No indication |
| 11/154 | 1/2b | 2011 | n-MN | Sepsis | Digestive diseases |
| 11/155 | 4b | 2011 | n-MN | Sepsis | No indication |
| 11/157 | 4b | 2011 | n-MN | Sepsis | Iatrogenic immunosuppression |
| 11/158 | 1/2a | 2011 | n-MN | Sepsis | Alcoholism |
| 11/159 | 1/2a | 2011 | n-MN | Sepsis | No indication |
| 11/160 | 1/2a | 2011 | MN | Stillbirth | No indication |
| 11/161 | 4b | 2011 | n-MN | Sepsis | Cancer |
| 11/165 | 1/2a | 2011 | n-MN | Sepsis | No indication |
| 11/166 | 1/2a | 2011 | n-MN | Sepsis | No indication |
| 11/168 | 1/2a | 2011 | n-MN | Sepsis | Cancer |
| 11/169 | 1/2a | 2011 | n-MN | Sepsis | Alcoholism |
| 11/178 | 1/2a | 2011 | n-MN | Sepsis | Digestive diseases |
| 11/179 | 4b | 2011 | n-MN | Sepsis | No indication |
| 11/187 | 1/2a | 2011 | n-MN | Unknown | No indication |
| 11/188 | 4b | 2011 | n-MN | Sepsis | Chronic kidney diseases |
| 11/189 | 1/2a | 2011 | n-MN | Sepsis | No indication |
| 11/190 | 4d | 2011 | n-MN | Sepsis | No indication |
| 11/193 | 1/2a | 2011 | n-MN | Sepsis | Immunosupression |
| 11/198 | 4b | 2011 | n-MN | Sepsis | Transplantation |
| 11/199 | 4b | 2011 | n-MN | Sepsis | No indication |
| 11/200 | 1/2a | 2011 | MN | Stillbirth | No indication |
| 11/201 | 1/2a | 2011 | n-MN | Peritonitis | Alcoholism |
| 11/202 | 1/2a | 2011 | n-MN | Sepsis | No indication |
| 11/204 | 4b | 2011 | n-MN | Premature birth | No indication |
| 11/212 | 4b | 2011 | n-MN | Sepsis | No indication |
| 11/215 | 4b | 2011 | n-MN | Sepsis | Respiratory diseases |
| 11/217 | 4b | 2011 | n-MN | Sepsis | Respiratory diseases |
| 11/222 | 4b | 2011 | n-MN | Sepsis | Respiratory diseases |
| 11/225 | 1/2a | 2011 | n-MN | Sepsis | Cancer |
| 11/229 | 1/2b | 2011 | MN | Sepsis | No indication |
| 11/230 | 4b | 2011 | n-MN | Sepsis | Cancer |
| 11/238 | 4b | 2011 | n-MN | Sepsis | Chronic kidney diseases |
| 11/239 | 1/2a | 2011 | n-MN | Sepsis | No indication |
| 11/242 | 4b | 2011 | n-MN | Sepsis | No indication |
| 11/243 | 4b | 2011 | MN | Sepsis | No indication |
| 11/267 | 4b | 2011 | n-MN | Sepsis | Alcoholism |
| 11/268 | 4b | 2011 | n-MN | Sepsis | Cancer |
| 11/269 | 4b | 2011 | n-MN | Sepsis | No indication |
| 11/308 | 1/2a | 2011 | n-MN | Sepsis | Respiratory diseases |
| 11/309 | 1/2a | 2011 | n-MN | Unknown | No indication |
| 11/317 | 4b | 2011 | n-MN | Sepsis | Cancer |
| 11/320 | 4b | 2011 | MN | Sepsis | No indication |
| 11/329 | 1/2c | 2011 | n-MN | Sepsis | No indication |
| 11/330 | 4b | 2011 | n-MN | Sepsis | No indication |
| 11/337 | 4b | 2011 | n-MN | Meningitis | No indication |
| 11/338 | 1/2b | 2011 | n-MN | Sepsis | No indication |
| 11/340 | 4b | 2011 | n-MN | Sepsis | Cancer |
| 12/015 | 4b | 2012 | MN | Stillbirth | No indication |
| 12/018 | 4b | 2012 | n-MN | Sepsis | No indication |
| 12/022 | 1/2a | 2012 | n-MN | Sepsis | Respiratory diseases |
| 12/023 | 4b | 2012 | MN | Stillbirth | No indication |
| 12/024 | 4b | 2012 | n-MN | Sepsis | No indication |
| 12/025 | 4b | 2012 | MN | Sepsis | No indication |
| 12/027 | 4b | 2012 | n-MN | Sepsis | Surgery |
| 12/028 | 4b | 2012 | MN | Unknown | No indication |
| 12/036 | 1/2a | 2012 | n-MN | Sepsis | No indication |
| 12/037 | 1/2a | 2012 | n-MN | Unknown | No indication |
| 12/038 | 4b | 2012 | n-MN | Meningitis + sepsis | No indication |
| 12/041 | 1/2b | 2012 | n-MN | Sepsis | No indication |
| 12/044 | 4b | 2012 | n-MN | Meningitis | No indication |
| 12/046 | 1/2b | 2012 | n-MN | Meningitis | No indication |
| 12/047 | 4b | 2012 | MN | Stillbirth | No indication |
| 12/061 | 1/2a | 2012 | n-MN | Meningitis | No indication |
| 12/064 | 4b | 2012 | n-MN | Sepsis | Cancer |
| 12/065 | 1/2b | 2012 | n-MN | Sepsis | No indication |
| 12/066 | 1/2a | 2012 | n-MN | Sepsis | Cancer |
| 12/067 | 1/2a | 2012 | n-MN | Sepsis | No indication |
| 12/072 | 1/2a | 2012 | n-MN | Sepsis | Digestive diseases |
| 12/073 | 4b | 2012 | n-MN | Sepsis | No indication |
| 12/075 | 4b | 2012 | MN | Sepsis | No indication |
| 12/082 | 4b | 2012 | n-MN | Sepsis | Chronic kidney diseases |
| 12/094 | 1/2a | 2012 | n-MN | Sepsis | No indication |
| 12/097 | 4b | 2012 | MN | Unknown | No indication |
| 12/098 | 1/2a | 2012 | n-MN | Sepsis | Cancer |
| 12/104 | 4b | 2012 | n-MN | Sepsis | Heart diseases |
| 12/105 | 4b | 2012 | n-MN | Sepsis | Cancer |
| 12/106 | 4b | 2012 | n-MN | Sepsis | Cancer |
| 12/108 | 3a | 2012 | n-MN | Unknown | No indication |
| 12/111 | 4b | 2012 | n-MN | Sepsis | Digestive diseases |
| 12/115 | 4b | 2012 | n-MN | Meningitis + sepsis | No indication |
| 12/117 | 1/2c | 2012 | n-MN | Sepsis | Respiratory diseases |
| 12/118 | 1/2a | 2012 | n-MN | Sepsis | No indication |
| 12/122 | 1/2a | 2012 | n-MN | Sepsis | Surgery |
| 12/125 | 1/2c | 2012 | n-MN | Sepsis | No indication |
| 12/142 | 1/2b | 2012 | n-MN | Meningitis | Cancer |
| 12/146 | 4b | 2012 | n-MN | Sepsis | Iatrogenic immunosuppression |
| 12/147 | 4b | 2012 | n-MN | Meningitis | Cancer |
| 12/148 | 1/2a | 2012 | n-MN | Sepsis | No indication |
| 12/149 | 1/2b | 2012 | n-MN | Sepsis | No indication |
| 12/150 | 1/2b | 2012 | n-MN | Sepsis | No indication |
| 12/159 | 4b | 2012 | n-MN | Sepsis | No indication |
| 12/163 | 1/2a | 2012 | n-MN | Meningitis | Digestive diseases |
| 12/167 | 4b | 2012 | n-MN | Meningitis | No indication |
| 12/168 | 1/2a | 2012 | n-MN | Sepsis | Cancer |
| 12/172 | 1/2b | 2012 | n-MN | Sepsis | No indication |
| 12/173 | 4b | 2012 | n-MN | Sepsis | No indication |
| 12/176 | 1/2a | 2012 | n-MN | Sepsis | Cancer |
| 12/178 | 1/2a | 2012 | n-MN | Sepsis | Cancer |
| 12/185 | 1/2a | 2012 | n-MN | Meningitis | No indication |
| 12/188 | 4b | 2012 | n-MN | Meningitis | No indication |
| 12/192 | 4b | 2012 | n-MN | Sepsis | Cancer |
| 12/200 | 4b | 2012 | n-MN | Sepsis | Cancer |
| 12/202 | 1/2a | 2012 | n-MN | Sepsis | Cancer |
| 12/203 | 1/2a | 2012 | n-MN | Sepsis | No indication |
| 12/204 | 4b | 2012 | n-MN | Sepsis | No indication |
| 12/206 | 1/2a | 2012 | n-MN | Meningitis + sepsis | Cancer |
| 12/211 | 4b | 2012 | n-MN | Sepsis | No indication |
| 12/229 | 4b | 2012 | n-MN | Sepsis | Cancer |
| 12/231 | 1/2a | 2012 | n-MN | Sepsis | Immunosupression |
| 12/232 | 1/2b | 2012 | n-MN | pustulosis | No indication |
| 13-15 | 1/2a | 2013 | n-MN | Sepsis | No indication |
| 13-18 | 1/2a | 2013 | n-MN | Sepsis | No indication |
| 13-2 | 4b | 2013 | MN | Stillbirth | No indication |
| 13-29 | 1/2a | 2013 | n-MN | Sepsis | Cancer |
| 13-37 | 1/2b | 2013 | n-MN | Sepsis | Cancer |
| 13-5 | 4b | 2013 | n-MN | Sepsis | No indication |
| S13BD00041 | 4b | 2013 | n-MN | Sepsis | No indication |
| S13BD00096 | 4b | 2013 | n-MN | Sepsis | No indication |
| S13BD00099 | 4b | 2013 | n-MN | Sepsis | Cancer |
| S13BD00153 | 1/2a | 2013 | n-MN | Sepsis | No indication |
| S13BD00158 | 1/2c | 2013 | n-MN | Sepsis | Immunosupression |
| S13BD00247 | 1/2a | 2013 | n-MN | Meningitis + sepsis | Immunosupression |
| S13BD00253 | 4b | 2013 | n-MN | Meningitis + sepsis | Cancer |
| S13BD00320 | 1/2b | 2013 | n-MN | Sepsis | Heart diseases |
| S13BD00328 | 1/2a | 2013 | n-MN | Peritonitis | No indication |
| S13BD00610 | 1/2a | 2013 | MN | Meningitis | No indication |
| S13BD00612 | 1/2a | 2013 | n-MN | Meningitis | No indication |
| S13BD00641 | 4b | 2013 | n-MN | Sepsis | No indication |
| S13BD00669 | 1/2a | 2013 | n-MN | Meningitis + sepsis | No indication |
| S13BD00832 | 1/2a | 2013 | n-MN | Sepsis | Cancer |
| S13BD00833 | 4b | 2013 | n-MN | Sepsis | Cancer |
| S13BD00849 | 1/2a | 2013 | n-MN | Sepsis | Cancer |
| S13BD01289 | 4b | 2013 | n-MN | Sepsis | Surgery |
| S13BD01473 | 4b | 2013 | n-MN | Sepsis | Surgery |
| S13BD01518 | 4b | 2013 | n-MN | Meningitis | No indication |
| S13BD01605 | 4b | 2013 | n-MN | Sepsis | Respiratory diseases |
| S13BD01697 | 4b | 2013 | n-MN | Meningitis | No indication |
| S13BD01838 | 4b | 2013 | n-MN | Sepsis | No indication |
| S13BD01923 | 4b | 2013 | n-MN | Sepsis | No indication |
| S13BD01929 | 1/2a | 2013 | n-MN | Peritonitis | Digestive diseases |
| S13BD01958 | 1/2a | 2013 | n-MN | Unknown | No indication |
| S13BD02030 | 1/2a | 2013 | n-MN | Sepsis | No indication |
| S13BD02095 | 4b | 2013 | n-MN | Meningitis + sepsis | Digestive diseases |
| S13BD02166 | 1/2a | 2013 | n-MN | Sepsis | No indication |
| S13BD02249 | 1/2b | 2013 | n-MN | Sepsis | Cancer |
| S13BD02290 | 1/2a | 2013 | n-MN | Sepsis | No indication |
| S13BD02291 | 1/2a | 2013 | n-MN | Sepsis | Cancer |
| S13BD02317 | 1/2a | 2013 | MN | Sepsis |  |
| S13BD02318 | 1/2a | 2013 | n-MN | Sepsis | Digestive diseases |
| S13BD02344 | 1/2a | 2013 | n-MN | Sepsis | Cancer |
| S13BD02415 | 1/2a | 2013 | n-MN | Sepsis | Cancer |
| S13BD02479 | 1/2a | 2013 | n-MN | Sepsis | Cancer |
| S13BD02501 | 1/2a | 2013 | n-MN | Sepsis | No indication |
| S13BD02601 | 1/2a | 2013 | n-MN | Sepsis | No indication |
| S13BD02693 | 3c | 2013 | n-MN | Sepsis | No indication |
| S13BD02700 | IVb | 2013 | n-MN | Sepsis | Heart diseases |
| S13BD02725 | 1/2a | 2013 | n-MN | Sepsis | Heart diseases |
| S13BD02767 | 1/2a | 2013 | n-MN | Sepsis | Transplantation |
| S13BD02992 | 1/2a | 2013 | n-MN | Sepsis | Cancer |
| S13BD03035 | 1/2b | 2013 | n-MN | Sepsis | No indication |
| S13BD03075 | 1/2a | 2013 | n-MN | Sepsis | Cancer |
| S13BD03097 | 1/2a | 2013 | n-MN | Sepsis | Chronic kidney diseases |
| S13BD03365 | 4b | 2013 | n-MN | Sepsis | Surgery |
| S13BD03580 | 1/2a | 2013 | n-MN | Meningitis + sepsis | Alcoholism |
| S13BD03610 | 1/2a | 2013 | n-MN | Sepsis | No indication |
| S13BD03713 | 4b | 2013 | n-MN | Meningitis | No indication |
| S13BD03845 | sp | 2013 | MN | Gastro-enteritis | No indication |
| S13BD03895 | 1/2a | 2013 | n-MN | Sepsis | Chronic kidney diseases |
| S13BD03980 | 1/2a | 2013 | n-MN | Sepsis | Cancer |
| S13BD04073 | 4b | 2013 | n-MN | Sepsis | No indication |
| S13BD04117 | 1/2b | 2013 | n-MN | Sepsis | Respiratory diseases |
| S13BD04118 | 4b | 2013 | n-MN | Peritonitis | Digestive diseases |
| S13BD04131 | 1/2a | 2013 | n-MN | Sepsis | No indication |
| S13BD04141 | 4b | 2013 | MN | Sepsis | No indication |
| S13BD04274 | 1/2a | 2013 | n-MN | Sepsis | No indication |
| S13BD04286 | 1/2a | 2013 | n-MN | Meningitis + sepsis | No indication |
| S13BD04289 | 4b | 2013 | MN | Premature birth | No indication |
| S13BD04355 | 1/2a | 2013 | n-MN | Sepsis | Respiratory diseases |
| S13BD04452 | 1/2a | 2013 | n-MN | Meningitis | Cancer |
| S14BD00001 | 1/2a | 2013 | n-MN | Sepsis | Immunosupression |
| S14BD00002 | 4b | 2013 | n-MN | Sepsis | No indication |
| S14BD00003 | 1/2a | 2013 | n-MN | Sepsis | Surgery |
| S14BD00138 | 1/2a | 2013 | n-MN | Sepsis | Cancer |
| S14BD00250 | 1/2a | 2014 | n-MN | Sepsis | No indication |
| S14BD00325 | 1/2a | 2014 | n-MN | Sepsis | Heart diseases |
| S14BD00326 | 1/2b | 2014 | n-MN | Sepsis | Cancer |
| S14BD00358 | 1/2a | 2014 | n-MN | Sepsis | No indication |
| S14BD00387 | 4b | 2014 | n-MN | Sepsis | Cancer |
| S14BD00429 | 1/2a | 2014 | n-MN | Sepsis | Digestive diseases |
| S14BD00463 | 1/2a | 2014 | n-MN | Sepsis | Cancer |
| S14BD00517 | 1/2a | 2014 | n-MN | Sepsis | No indication |
| S14BD00663 | 4b | 2014 | n-MN | Sepsis | Digestive diseases |
| S14BD00685 | 1/2a | 2014 | n-MN | Sepsis | Cancer |
| S14BD00735 | 1/2a | 2014 | n-MN | Sepsis | No indication |
| S14BD00903 | 4b | 2014 | n-MN | Sepsis | Digestive diseases |
| S14BD01004 | 1/2a | 2014 | n-MN | Sepsis | No indication |
| S14BD01104 | 1/2a | 2014 | n-MN | Sepsis | Heart diseases |
| S14BD01181 | 4b | 2014 | n-MN | Sepsis | No indication |
| S14BD01200 | 1/2a | 2014 | n-MN | Sepsis | No indication |
| S14BD01259 | 1/2a | 2014 | n-MN | Sepsis | Cancer |
| S14BD01271 | 1/2a | 2014 | n-MN | Sepsis | Cancer |
| S14BD01292 | 1/2a | 2014 | n-MN | Sepsis | Cancer |
| S14BD01307 | 1/2a | 2014 | n-MN | Meningitis | Digestive diseases |
| S14BD01674 | 1/2a | 2014 | n-MN | Meningitis + sepsis | Iatrogenic immunosuppression |
| S14BD01686 | 4b | 2014 | n-MN | Sepsis | Cancer |
| S14BD01696 | 1/2a | 2014 | n-MN | Sepsis | Transplantation |
| S14BD01752 | 1/2a | 2014 | n-MN | Sepsis | Respiratory diseases |
| S14BD01846 | 1/2a | 2014 | n-MN | Sepsis | Chronic kidney diseases |
| S14BD02028 | 4b | 2014 | n-MN | Sepsis | No indication |
| S14BD02040 | 1/2a | 2014 | MN | Premature birth | No indication |
| S14BD02128 | 4b | 2014 | n-MN | Sepsis | Respiratory diseases |
| S14BD02234 | 1/2c | 2014 | n-MN | Sepsis | No indication |
| S14BD02423 | 4b | 2014 | n-MN | Sepsis | No indication |
| S14BD02538 | 1/2a | 2014 | n-MN | Meningitis + sepsis | No indication |
| S14BD02561 | 1/2c | 2014 | n-MN | Sepsis | Cancer |
| S14BD02637 | 1/2a | 2014 | n-MN | Sepsis | Cancer |
| S14BD02716 | 1/2a | 2014 | n-MN | Meningitis | No indication |
| S14BD02717 | 1/2a | 2014 | n-MN | Meningitis | No indication |
| S14BD02718 | 1/2a | 2014 | n-MN | Sepsis | Digestive diseases |
| S14BD02863 | 1/2b | 2014 | n-MN | Meningitis | Digestive diseases |
| S14BD02947 | 1/2a | 2014 | n-MN | Sepsis | Chronic kidney diseases |
| S14BD02955 | 1/2a | 2014 | n-MN | Sepsis | No indication |
| S14BD02956 | 4b | 2014 | n-MN | Unknown | No indication |
| S14BD02965 | 1/2a | 2014 | n-MN | Sepsis | Cancer |
| S14BD03044 | 1/2b | 2014 | n-MN | Unknown | Digestive diseases |
| S14BD03045 | 4b | 2014 | n-MN | Sepsis | No indication |
| S14BD03108 | 4b | 2014 | n-MN | Unknown | No indication |
| S14BD03109 | 4b | 2014 | n-MN | Sepsis | No indication |
| S14BD03177 | 1/2a | 2014 | n-MN | Sepsis | Cancer |
| s14BD03301 | 1/2a | 2014 | n-MN | Sepsis | Cancer |
| s14BD03405 | 1/2c | 2014 | n-MN | Sepsis | No indication |
| s14BD03406 | 1/2a | 2014 | n-MN | Meningitis + sepsis | Alcoholism |
| s14BD03419 | 4d | 2014 | n-MN | Sepsis | Cancer |
| s14BD03439 | 1/2a | 2014 | n-MN | Sepsis | Heart diseases |
| s14BD03505 | 1/2a | 2014 | n-MN | Sepsis | No indication |
| s14BD03561 | 1/2a | 2014 | n-MN | Sepsis | Iatrogenic immunosuppression |
| s14BD03562 | 4b | 2014 | n-MN | Sepsis | Cancer |
| s14BD03563 | 1/2a | 2014 | n-MN | Sepsis | Cancer |
| s14BD03589 | 1/2a | 2014 | n-MN | Sepsis | Heart diseases |
| s14BD03659 | 4b | 2014 | n-MN | Meningitis | No indication |
| s14BD03672 | 4b | 2014 | n-MN | Unknown | No indication |
| s14BD03734 | Autoagglutinable | 2014 | n-MN | Sepsis | No indication |
| s14BD03865 | 1/2c | 2014 | n-MN | Unknown | No indication |
| s14BD03866 | 4b | 2014 | MN | Stillbirth | No indication |
| s14BD03916 | 1/2a | 2014 | n-MN | Sepsis | Cancer |
| s14BD03935 | 4b | 2014 | n-MN | Sepsis | Cancer |
| s14BD04035 | 1/2a | 2014 | MN | Premature birth | No indication |
| s14BD04402 | 1/2a | 2014 | MN | Unknown | No indication |
| s14BD04569 | 4b | 2014 | n-MN | Peritonitis | Cancer |
| s14BD04576 | 4b | 2014 | n-MN | Sepsis | No indication |
| s14BD04775 | 1/2a | 2014 | n-MN | Sepsis | Cancer |
| s14BD04817 | 1/2a | 2014 | n-MN | Sepsis | Alcoholism |
| s14BD04941 | 4b | 2014 | MN | Sepsis | Immunosupression |
| s14BD04942 | 1/2b | 2014 | n-MN | Sepsis | Cancer |
| s14BD05052 | 1/2a | 2014 | n-MN | Meningitis + sepsis | No indication |
| s14BD05132 | 4b | 2014 | n-MN | Meningitis + sepsis | Cancer |
| s14BD05174 | 1/2a | 2014 | n-MN | Sepsis | No indication |
| s14BD05284 | 1/2b | 2014 | n-MN | Sepsis | Digestive diseases |
| s14BD05360 | 4b | 2014 | n-MN | Meningitis + sepsis | No indication |
| s14BD05426 | 1/2a | 2014 | n-MN | Sepsis | No indication |
| S14BD05524 | 1/2a | 2014 | n-MN | Sepsis | No indication |
| S14BD05525 | 1/2b | 2014 | n-MN | Sepsis | Cancer |
| S14BD05536 | 1/2a | 2014 | n-MN | Sepsis | Respiratory diseases |
| S14BD05549 | 1/2a | 2014 | n-MN | Sepsis | No indication |
| S14BD05602 | 4b | 2014 | n-MN | Sepsis | Digestive diseases |
| S15BD00001 | 1/2a | 2014 | n-MN | Sepsis | No indication |
