# Supplementary material for: Diversity of Listeria monocytogenes Strains of Clinical and Food Chain Origins in Belgium between 1985 and 2014
Source: PLoS One. 2016 Oct 10;11(10):e0164283. doi: 10.1371/journal.pone.0164283 (PMC5056710; doi:10.1371/journal.pone.0164283)
Supplement: S4 Table — (DOCX) [file pone.0164283.s004.docx]

**S4 Table. List of food Listeria isolates used in this study with their main characteristics**

| **ID** | **Serotype** | **Year** | **Matrix** |
| --- | --- | --- | --- |
| LM09003 | 1/2a | 2009 | Meat and meat products |
| LM09007 | 1/2a | 2009 | Meat and meat products |
| LM09008 | 1/2a | 2009 | Meat and meat products |
| LM09015 | 1/2a | 2009 | Meat and meat products |
| LM09032 | 1/2a | 2009 | Ready to eat meal |
| LM09047 | 1/2a | 2009 | Meat and meat products |
| LM09048 | 1/2a | 2009 | Meat and meat products |
| LM09052 | 1/2a | 2009 | Milk and milk products |
| LM09053 | 1/2a | 2009 | Meat and meat products |
| LM09054 | 1/2a | 2009 | Fish and seafood |
| LM09056 | 1/2a | 2009 | Meat and meat products |
| LM09066 | 1/2a | 2009 | Meat and meat products |
| LM09067 | 1/2a | 2009 | Meat and meat products |
| LM09078 | 1/2a | 2009 | Fruits and vegetables |
| LM09079 | 1/2a | 2009 | Fruits and vegetables |
| LM09082 | 1/2a | 2009 | Ready to eat meal |
| LM09085 | 1/2a | 2009 | Meat and meat products |
| LM09252 | 1/2a | 2009 | Meat and meat products |
| LM09254 | 1/2a | 2009 | Meat and meat products |
| LM09255 | 1/2a | 2009 | Meat and meat products |
| LM10020 | 1/2a | 2010 | Meat and meat products |
| LM10023 | 1/2a | 2010 | Fish and seafood |
| LM10026 | 1/2a | 2010 | Meat and meat products |
| LM10057 | 1/2a | 2010 | Meat and meat products |
| LM10069 | 1/2a | 2010 | Milk and milk products |
| LM10070 | 1/2a | 2010 | Meat and meat products |
| LM10076 | 1/2a | 2010 | Meat and meat products |
| LM10082 | 1/2a | 2010 | Milk and milk products |
| LM10083 | 1/2a | 2010 | Milk and milk products |
| LM10094 | 1/2a | 2010 | Meat and meat products |
| LM10104 | 1/2a | 2010 | Ready to eat meal |
| LM10156 | 1/2a | 2010 | Milk and milk products |
| LM10161 | 1/2a | 2010 | Fish and seafood |
| LM10200 | 1/2a | 2010 | Meat and meat products |
| LM10210 | 1/2a | 2010 | Fish and seafood |
| LM10240 | 1/2a | 2010 | Meat and meat products |
| LM11001 | 1/2a | 2011 | Fish and seafood |
| LM11002 | 1/2a | 2011 | Meat and meat products |
| LM11020 | 1/2a | 2011 | Meat and meat products |
| LM11025 | 1/2a | 2011 | Fish and seafood |
| LM11028 | 1/2a | 2011 | Meat and meat products |
| LM11032 | 1/2a | 2011 | Fish and seafood |
| LM11037 | 1/2a | 2011 | Meat and meat products |
| LM11043 | 1/2a | 2011 | Meat and meat products |
| LM11049 | 1/2a | 2011 | Ready to eat meal |
| LM11066 | 1/2a | 2011 | Meat and meat products |
| LM11097 | 1/2a | 2011 | Fish and seafood |
| LM11101 | 1/2a | 2011 | Fish and seafood |
| LM11109 | 1/2a | 2011 | Milk and milk products |
| LM11119 | 1/2a | 2011 | Meat and meat products |
| LM11123 | 1/2a | 2011 | Fish and seafood |
| LM11127 | 1/2a | 2011 | Meat and meat products |
| LM11128 | 1/2a | 2011 | Meat and meat products |
| LM11130 | 1/2a | 2011 | Meat and meat products |
| LM11137 | 1/2a | 2011 | Fish and seafood |
| LM11145 | 1/2a | 2011 | Milk and milk products |
| LM11151 | 1/2a | 2011 | Meat and meat products |
| LM11177 | 1/2a | 2011 | Ready to eat meal |
| LM11186 | 1/2a | 2011 | Unknown |
| LM11203 | 1/2a | 2011 | Ready to eat meal |
| LM11219 | 1/2a | 2011 | Meat and meat products |
| LM11231 | 1/2a | 2011 | Fish and seafood |
| LM11232 | 1/2a | 2011 | Fish and seafood |
| LM11233 | 1/2a | 2011 | Fish and seafood |
| LM11251 | 1/2a | 2011 | Fish and seafood |
| LM11252 | 1/2a | 2011 | Fish and seafood |
| LM11255 | 1/2a | 2011 | Meat and meat products |
| LM11272 | 1/2a | 2011 | Fish and seafood |
| LM11277 | 1/2a | 2011 | Fish and seafood |
| LM11279 | 1/2a | 2011 | Meat and meat products |
| LM11284 | 1/2a | 2011 | Fish and seafood |
| LM11295 | 1/2a | 2011 | Fish and seafood |
| LM11299 | 1/2a | 2011 | Meat and meat products |
| LM11301 | 1/2a | 2011 | Fish and seafood |
| LM11305 | 1/2a | 2011 | Fish and seafood |
| LM11307 | 1/2a | 2011 | Meat and meat products |
| LM11324 | 1/2a | 2011 | Milk and milk products |
| LM11331 | 1/2a | 2011 | Milk and milk products |
| LM11332 | 1/2a | 2011 | Meat and meat products |
| LM11334 | 1/2a | 2011 | Meat and meat products |
| LM11335 | 1/2a | 2011 | Fish and seafood |
| LM11339 | 1/2a | 2011 | Unknown |
| LM12002 | 1/2a | 2012 | Meat and meat products |
| LM12003 | 1/2a | 2012 | Meat and meat products |
| LM12004 | 1/2a | 2012 | Fish and seafood |
| LM12009 | 1/2a | 2012 | Ready to eat meal |
| LM12016 | 1/2a | 2012 | Fish and seafood |
| LM12039 | 1/2a | 2012 | Meat and meat products |
| LM12058 | 1/2a | 2012 | Milk and milk products |
| LM12062 | 1/2a | 2012 | Ready to eat meal |
| LM12069 | 1/2a | 2012 | Meat and meat products |
| LM12083 | 1/2a | 2012 | Meat and meat products |
| LM12090 | 1/2a | 2012 | Milk and milk products |
| LM12092 | 1/2a | 2012 | Fish and seafood |
| LM12096 | 1/2a | 2012 | Meat and meat products |
| LM12102 | 1/2a | 2012 | Fruits and vegetables |
| LM12107 | 1/2a | 2012 | Fish and seafood |
| LM12109 | 1/2a | 2012 | Fruits and vegetables |
| LM12110 | 1/2a | 2012 | Fruits and vegetables |
| LM12113 | 1/2a | 2012 | Meat and meat products |
| LM12114 | 1/2a | 2012 | Meat and meat products |
| LM12127 | 1/2a | 2012 | Fruits and vegetables |
| LM12129 | 1/2a | 2012 | Fruits and vegetables |
| LM12138 | 1/2a | 2012 | Fish and seafood |
| LM12144 | 1/2a | 2012 | Meat and meat products |
| LM12153 | 1/2a | 2012 | Milk and milk products |
| LM12164 | 1/2a | 2012 | Fish and seafood |
| LM12171 | 1/2a | 2012 | Meat and meat products |
| LM12184 | 1/2a | 2012 | Meat and meat products |
| LM12189 | 1/2a | 2012 | Milk and milk products |
| LM12207 | 1/2a | 2012 | Meat and meat products |
| LM12208 | 1/2a | 2012 | Milk and milk products |
| LM12213 | 1/2a | 2012 | Milk and milk products |
| LM12219 | 1/2a | 2012 | Milk and milk products |
| LM12220 | 1/2a | 2012 | Milk and milk products |
| LM12221 | 1/2a | 2012 | Milk and milk products |
| LM12223 | 1/2a | 2012 | Meat and meat products |
| LM12224 | 1/2a | 2012 | Meat and meat products |
| LM12225 | 1/2a | 2012 | Fish and seafood |
| LM13017 | 1/2a | 2013 | Fish and seafood |
| LM13019 | 1/2a | 2013 | Milk and milk products |
| LM13022 | 1/2a | 2013 | Unknown |
| LM13025 | 1/2a | 2013 | Milk and milk products |
| LM13028 | 1/2a | 2013 | Milk and milk products |
| S13BD00103 | 1/2a | 2013 | Meat and meat products |
| S13BD00142 | 1/2a | 2013 | Fish and seafood |
| S13BD00287 | 1/2a | 2013 | Meat and meat products |
| S13BD00315 | 1/2a | 2013 | Unknown |
| S13BD00364 | 1/2a | 2013 | Meat and meat products |
| S13BD00393 | 1/2a | 2013 | Milk and milk products |
| S13BD00454 | 1/2a | 2013 | Unknown |
| S13BD00469 | 1/2a | 2013 | Fish and seafood |
| S13BD00563 | 1/2a | 2013 | Fish and seafood |
| S13BD00626 | 1/2a | 2013 | Unknown |
| S13BD00768 | 1/2a | 2013 | Unknown |
| S13BD00859 | 1/2a | 2013 | Meat and meat products |
| S13BD01204 | 1/2a | 2013 | Fish and seafood |
| S13BD01277 | 1/2a | 2013 | Unknown |
| S13BD01357 | 1/2a | 2013 | Meat and meat products |
| S13BD01358 | 1/2a | 2013 | Meat and meat products |
| S13BD01479 | 1/2a | 2013 | Milk and milk products |
| S13BD01585 | 1/2a | 2013 | Fish and seafood |
| S13BD01710 | 1/2a | 2013 | Milk and milk products |
| S13BD01728 | 1/2a | 2013 | Milk and milk products |
| S13BD01842 | 1/2a | 2013 | Fruits and vegetables |
| S13BD02435 | 1/2a | 2013 | Meat and meat products |
| S13BD02796 | 1/2a | 2013 | Fruits and vegetables |
| S13BD02861 | 1/2a | 2013 | Meat and meat products |
| S13BD04163 | 1/2a | 2013 | Meat and meat products |
| S13BD04236 | 1/2a | 2013 | Meat and meat products |
| S13BD04480 | 1/2a | 2013 | Milk and milk products |
| S13FP02507 | 1/2a | 2013 | Meat and meat products |
| S13FP02508 | 1/2a | 2013 | Meat and meat products |
| S13FP02820 | 1/2a | 2013 | Meat and meat products |
| S14BD00289 | 1/2a | 2014 | Fish and seafood |
| S14BD00395 | 1/2a | 2014 | Fish and seafood |
| S14BD00564 | 1/2a | 2014 | Meat and meat products |
| S14BD00621 | 1/2a | 2014 | Milk and milk products |
| S14BD00698 | 1/2a | 2014 | Fish and seafood |
| S14BD00762 | 1/2a | 2014 | Milk and milk products |
| S14BD00862 | 1/2a | 2014 | Milk and milk products |
| S14BD00997 | 1/2a | 2014 | Milk and milk products |
| S14BD01203 | 1/2a | 2014 | Meat and meat products |
| S14BD01388 | 1/2a | 2014 | Milk and milk products |
| S14BD01618 | 1/2a | 2014 | Meat and meat products |
| S14BD01903 | 1/2a | 2014 | Fish and seafood |
| S14BD01977 | 1/2a | 2014 | Meat and meat products |
| S14BD02038 | 1/2a | 2014 | Meat and meat products |
| S14BD02061 | 1/2a | 2014 | Ready to eat meal |
| S14BD02225 | 1/2a | 2014 | Meat and meat products |
| S14BD02322 | 1/2a | 2014 | Ready to eat meal |
| S14BD02416 | 1/2a | 2014 | Meat and meat products |
| S14BD02844 | 1/2a | 2014 | Milk and milk products |
| S14BD03277 | 1/2a | 2014 | Milk and milk products |
| S14BD03468 | 1/2a | 2014 | Milk and milk products |
| LM09009 | 4b | 2009 | Meat and meat products |
| LM09010 | 4b | 2009 | Meat and meat products |
| LM09029 | 4b | 2009 | Meat and meat products |
| LM09034 | 4b | 2009 | Meat and meat products |
| LM090342 | 4b | 2009 | Meat and meat products |
| LM09035 | 4b | 2009 | Meat and meat products |
| LM09036 | 4b | 2009 | Meat and meat products |
| LM09043 | 4b | 2009 | Fish and seafood |
| LM09045 | 4b | 2009 | Fish and seafood |
| LM09058 | 4b | 2009 | Fish and seafood |
| LM090582 | 4b | 2009 | Fish and seafood |
| LM09059 | 4b | 2009 | Fish and seafood |
| LM09060 | 4b | 2009 | Fish and seafood |
| LM09061 | 4b | 2009 | Fish and seafood |
| LM09062 | 4b | 2009 | Fish and seafood |
| LM09065 | 4b | 2009 | Fish and seafood |
| LM09074 | 4b | 2009 | Meat and meat products |
| LM09086 | 4b | 2009 | Meat and meat products |
| LM09087 | 4b | 2009 | Fruits and vegetables |
| LM09257 | 4b | 2009 | Milk and milk products |
| LM09258 | 4b | 2009 | Milk and milk products |
| LM09260 | 4b | 2009 | Milk and milk products |
| LM10007 | 4b | 2010 | Fish and seafood |
| LM10034 | 4b | 2010 | Milk and milk products |
| LM10149 | 4b | 2010 | Milk and milk products |
| LM10155 | 4b | 2010 | Meat and meat products |
| LM10175 | 4b | 2010 | Ready to eat meal |
| LM10207 | 4b | 2010 | Meat and meat products |
| LM11011 | 4b | 2011 | Milk and milk products |
| LM11030 | 4b | 2011 | Milk and milk products |
| LM11048 | 4b | 2011 | Ready to eat meal |
| LM11052 | 4b | 2011 | Milk and milk products |
| LM11062 | 4b | 2011 | Meat and meat products |
| LM11117 | 4b | 2011 | Milk and milk products |
| LM11149 | 4b | 2011 | Meat and meat products |
| LM11196 | 4b | 2011 | Fish and seafood |
| LM11273 | 4b | 2011 | Meat and meat products |
| LM11275 | 4b | 2011 | Milk and milk products |
| LM11286 | 4b | 2011 | Fish and seafood |
| LM12005 | 4b | 2012 | Ready to eat meal |
| LM12006 | 4b | 2012 | Meat and meat products |
| LM12014 | 4b | 2012 | Meat and meat products |
| LM12026 | 4b | 2012 | Ready to eat meal |
| LM12085 | 4b | 2012 | Milk and milk products |
| LM12093 | 4b | 2012 | Ready to eat meal |
| LM12128 | 4b | 2012 | Milk and milk products |
| LM12137 | 4b | 2012 | Milk and milk products |
| LM12154 | 4b | 2012 | Ready to eat meal |
| LM12155 | 4b | 2012 | Meat and meat products |
| LM12158 | 4b | 2012 | Ready to eat meal |
| LM12182 | 4b | 2012 | Unknown |
| LM12186 | 4b | 2012 | Ready to eat meal |
| LM13006 | 4b | 2013 | Meat and meat products |
| LM13030 | 4b | 2013 | Milk and milk products |
| S13BD00007 | 4b | 2013 | Ready to eat meal |
| S13BD00190 | 4b | 2013 | Fish and seafood |
| S13BD04146 | 4b | 2013 | Fish and seafood |
| S14BD00296 | 4b | 2014 | Meat and meat products |
| S14BD01659 | 4b | 2014 | Meat and meat products |
| S14BD01902 | 4b | 2014 | Fish and seafood |
| S14BD02035 | 4b | 2014 | Meat and meat products |
| S14BD02037 | 4b | 2014 | Milk and milk products |
| S14BD02227 | 4b | 2014 | Milk and milk products |
| S14BD02281 | 4b | 2014 | Meat and meat products |
| S14BD04025 | 4b | 2014 | Meat and meat products |
| S14BD04120 | 4b | 2014 | Meat and meat products |
| S14BD04381 | 4b | 2014 | Meat and meat products |
