# Supplementary material for: Diversity of Listeria monocytogenes Strains of Clinical and Food Chain Origins in Belgium between 1985 and 2014
Source: PLoS One. 2016 Oct 10;11(10):e0164283. doi: 10.1371/journal.pone.0164283 (PMC5056710; doi:10.1371/journal.pone.0164283)
Supplement: S5 Table — (DOCX) [file pone.0164283.s005.docx]

**S5 Table. List of veterinary Listeria isolates used in this study with their main characteristics**

| **ID** | **Serotype** | **Origin** | **Specimen** |
| --- | --- | --- | --- |
| S15BD01072 | 4b | Sheep | Brain |
| S15BD01073 | 1/2a | Sheep | Brain |
| S15BD01074 | 4b | Goat | Brain |
| S15BD01075 | 4b | Cow | Brain |
| S15BD01076 | 1/2a | Horse | Pus |
| S15BD01079 | 1/2a | Sheep | Unknown |
| S15BD01080 | 1/2a | Reptile | Heart |
| S15BD01082 | 1/2a | Sheep | Unknown |
| S15BD01083 | 4b | Cow | Unknown |
| S15BD01084 | 4b | Sheep | Brain |
| S15BD01085 | 4b | Goat | Unknown |
| S15BD01087 | 1/2a | Bird | Liver |
| S15BD01089 | 4b | Cow | Brain |
| S15BD01090 | 4b |  | Brain |
| S15BD01091 | 4b | Rodent | Liver |
| S15BD01095 | 4b | Bird | Liver |
| S15BD01096 | 4b | Goat | Brain |
| S15BD01097 | 4b | Cow | Brain |
| S15BD01098 | 4b | Sheep | Brain |
| S15BD01099 | 4b | Sheep | Brain |
| S15BD01100 | 4b | Goat | Blood |
| S15BD01102 | 4b | Reptile | Liver |
