# Supplementary material for: Diversity of Listeria monocytogenes Strains of Clinical and Food Chain Origins in Belgium between 1985 and 2014
Source: PLoS One. 2016 Oct 10;11(10):e0164283. doi: 10.1371/journal.pone.0164283 (PMC5056710; doi:10.1371/journal.pone.0164283)
Supplement: S6 Table — (N = Number of cases). (DOCX) [file pone.0164283.s006.docx]

**S6 Table. Seasonal repartition of Listeriosis (n-MN and MN) detected in Belgium since 2000. (N= Number of cases)**

|  | **N** | **%** |
| --- | --- | --- |
| Winter (Dec-Feb) | 193 | 22.4 |
| Spring (Mar-May) | 173 | 20.0 |
| Summer (Jun-Aug) | 250 | 29.0 |
| Autumn (Sep-Nov) | 247 | 28.6 |
